# Recent Progress in Flexible Wearable Sensors Utilizing Conductive Hydrogels for Sports Applications: Characteristics, Mechanisms, and Modification Strategies

**DOI:** 10.3390/gels11080589

**Published:** 2025-07-30

**Authors:** Jie Wu, Jingya Hong, Xing Gao, Yutong Wang, Wenyan Wang, Hongchao Zhang, Jaeyoung Park, Weiquan Shi, Wei Guo

**Affiliations:** 1 College of Art and Physical Education, Kyungil University, Gyeongsan-si 38428, Republic of Korea; wujie@hrbipe.edu.cn (J.W.);; 2 School of Management, Xiamen University, Xiamen 361005, China; 3 College of Sports and Human Sciences, Graduate School, Harbin Sport University, Harbin 150008, China

**Keywords:** conductive hydrogel, flexible wearable sensor, sensing mechanism, sports monitoring application

## Abstract

Conductive hydrogels demonstrate substantial potential for flexible wearable sensors in motion monitoring, owing to their unique physicochemical properties; however, current implementations still confront persistent challenges in long-term stability, sensitivity, response speed, and detection limits under complex dynamic conditions, which material innovations are urgently required to resolve. Consequently, this paper comprehensively reviews the recent advancements in conductive hydrogel-based flexible wearable sensors for sports applications. The paper examines the conductivity, self-adhesion, self-repair, and biocompatibility of conductive hydrogels, along with detailed analyses of their working principles in resistance, capacitance, piezoelectric, and battery-based sensing mechanisms. Additionally, the paper summarizes innovative strategies to enhance sensor performance through polymer blending, polyelectrolyte doping, inorganic salt doping, and nanomaterial integration. Furthermore, the paper highlights the latest applications of conductive hydrogel flexible wearable sensors in human motion monitoring, electrophysiological signal detection, and electrochemical biosignal monitoring. Finally, the paper provides an in-depth discussion of the advantages and limitations of existing technologies, offering valuable insights and new perspectives for future research directions.

## 1. Introduction

In contemporary competitive sports, scientifically customized and personalized training programs have become increasingly crucial for achieving exceptional athletic performance. The advancement of sensor technology, especially its miniaturization, intelligentization, and functional diversification, has furnished innovative tools for motion analysis and substantially broadened its application scope in numerous sports fields [1]. Flexible wearable sensors have emerged as pivotal tools for real-time monitoring of physiological data related to human movement, attributable to their rapid response, high sensitivity, and flexibility. This capability not only augments the accuracy and efficiency of athletic performance but also significantly reduces the risk of sports injuries [2].

Among these developments, conductive hydrogels have garnered considerable attention, attributed to their distinctive three-dimensional network structure and high water content. Their exceptional properties, such as tensile strength, flexibility, self-adhesiveness, conductivity, and biocompatibility, make them ideal materials for flexible wearable sensors [3]. Unlike traditional rigid sensors, conductive hydrogel flexible wearable sensors (CH-FWSs) possess unique value in physical advantages, chemical stability, biocompatibility, and applicability [4]. For example, their high water content and three-dimensional structure afford exceptional flexibility and stretchability, enabling them to closely conform to the body’s complex contours. Even during large-amplitude motions or in complex areas such as fingers or knees, they maintain stable attachment and prevent signal interference [4]. Recent research has predominantly concentrated on modifying hydrogel networks through functional groups, additives, and fillers—including inorganic salt doping, nanomaterial integration, and conductive polymer or polyelectrolyte combinations—to enhance sensor stability, adaptability, and multifunctionality in human-motion monitoring [5,6]. For instance, integrating nano-cellulose into a polyvinyl alcohol/polyacrylamide multi-functional ionic conductive hydrogel substantially improves its tensile and electrical properties [7]. As demonstrated in Bao et al.’s research [8], hydrogels show significant potential in developing soft, flexible, and durable electronic devices capable of meeting human needs and enabling continuous physiological parameter monitoring. Their work has made pioneering contributions to applying hydrogels in stretchable electronics, paving the way for wearable sensor development. This innovative design not only improves material mechanical properties but also enhances its adaptability and stability in complex motion environments, accelerating progress across the entire field.

Despite extensive research on CH-FWSs in sports monitoring, their practical applications still face substantial challenges. The durability, accuracy, and stability of CH-FWSs under varying environmental conditions—such as humidity and temperature fluctuations—remain pressing issues that require urgent addressing [9]. Traditional conductive hydrogels often suffer damage, hardening, or collapse under extreme temperature and humidity conditions, resulting in sensor performance degradation or failure [10]. Furthermore, CH-FWSs encounter challenges in long-term stability and adaptability to various physical conditions, such as rapid motion, sweating, or humid environments [11]. While most studies indicate that CH-FWSs can be utilized for human motion monitoring, a comprehensive summary of their specific applications, challenges, and potential solutions in this domain remains absent.

To address the research gap in systematic reviews of CH-FWSs in sports applications, this review aims to investigate the key advancements of CH-FWSs and analyze their potential in sports monitoring. This review commences by elaborating on the key characteristics of conductive hydrogels, including conductivity, adhesion, self-healing, and biocompatibility, and conducts an in-depth examination of their conductive mechanisms to enhance researchers’ comprehensive understanding of conductive hydrogel flexible strain sensors. Subsequently, this review discusses the latest improvement strategies for CH-FWSs, including material modification and structural optimization, to achieve sensor performance featuring high conductivity, tensile strength, a wide strain working range, enhanced sensitivity, and a rapid response. Furthermore, this review delves into the specific application scenarios of CH-FWSs in human motion monitoring, motion electrophysiological signal detection, and motion electrochemical biosignal monitoring. Finally, this review summarizes the current applications of conductive hydrogel flexible wearable sensors in motion monitoring and explores their future development directions, providing novel insights and guidance for the advancement of conductive hydrogel flexible strain sensors in motion monitoring.

## 2. Characteristics of Conductive Hydrogels

The exceptional performance of conductive hydrogels not only highlights their vast potential in sensing technologies but also lays a foundation for comprehensively exploring their performance attributes and underlying mechanisms. This, in turn, robustly drives the design and evolution of next-generation flexible sensors leveraging conductive hydrogels. This section systematically categorizes and evaluates key characteristics of conductive hydrogel-based flexible wearable sensors documented in the literature, such as conductivity, adhesion, biocompatibility, and self-healing capabilities, to offer critical insights for material selection and innovative advancement in this domain. Table 1 comprehensively encapsulates conductive modes, performance traits, and real-world applications of diverse conductive hydrogels, while distinctly highlighting interdependencies among pivotal factors like material categories, primary characteristics, performance metrics, and application scenarios.

**Table 1:** Performance characteristics of hydrogels categorized by various sensing mechanisms.

Sensing Mechanism	Materials	Mechanical Strength	Freeze Tolerance	Tensile Strain	Sensitivity (GF)	Conductivity	Performance Characteristics	Application Scenarios	Ref.
Resistance mode sensors	A, AM, KPS, LiCl	0.206 MPa	−37 °C	680%	3.12	R = 1.35	Frost resistance, elastic recoverability, toughness, adhesion, self-healing properties	Monitoring human fingers, wrists, and elbows during exercise	[12]
	PVA, CNTs, GO	-	-	900%	52.7	-	Re-plasticity, self-healing properties, elastic strain (900%), mechanical pressure (10 kPa)	Wearable electronic devices, soft robots	[13]
	HPMC, AA, AM, TP, Al^3+^	0.032 MPa	-	2225%	5.13	R = 3.03	Stretchability (GF5.13), adhesion, self-healing performance (99.4%)	Monitoring joint and vocal cord vibrations during various movements	[14]
	CS, OHA, HPMC, PAA, TA, Al^3+^	0.0338 MPa	-	3168%	<1	R = 2.33	Self-healing performance (95.5%), high transparency (98.5% transmittance), stretchability (GF4.12), adhesion	Monitoring joint bending movements, swallowing, and speaking during exercise	[15]
	PAM, CA, EtOH, DN	0.581 MPa	-	1545%	1.53	R = 1.53	Biocompatibility, stretchability, high sensitivity (GF1.63), adhesion	Sports wearable devices and human–machine interface systems	[16]
	Starch, PHMB, glycerol, PVA	0.209 MPa	−20 °C	406%	1.81	-	Stretching resistance (GF 3.28), frost resistance (−128.9 °C), antibacterial properties, water retention properties	Monitoring various human movements, including speaking, finger bending, and knee bending	[17]
	NaSS, DMC, MBAA, NaSS/DMC	0.06 MPa	-	418%	2.9	-	Mechanical properties, self-healing properties, stretchability (GF 2.9), high cycling stability	Monitoring human movements, including bending of fingers, elbows, and knees	[18]
	MBAA, FeCl_3_·6H_2_O, APS, EDTA-2Na, MLA	0.063 MPa	-	556%	2.6	-	Stretchability (556%), high sensitivity, self-healing performance (99%).	Monitoring human movements, including finger, wrist, and elbow bending, throat swallowing, and pulse	[19]
	PVA, SA, NaCl, TENG	0.58 MPa	-	-	-	R = 4.1	Tensile strength (0.58 MPa), mechanical properties, conductivity	Human motion monitoring, pedometer, step frequency meter	[20]
	SA, PANI, GO, An, CaCl_2_	10.32 MPa	-	176%	-	R = 0.5	Cyclic stretchability, high sensitivity, repeatability, swelling, mechanical properties	Monitoring human movements, including palms, elbows, and knees	[21]
	PVA, O-CMCS, AgNPs, PDO, ZnSO_4_	2.2 MPa	−60 °C	407%	2.26	R = 1.4	High strength (2.2 MPa), frost resistance, ductility, high toughness, self-healing ability	Monitoring human motion at low temperatures, including body movement and bioelectric signals	[22]
	PVA, SA, CNF, MXene, GO	0.21 MPa	-	611.5%	2.77	-	Stretchability (GF 2.77), sensitivity, high toughness, temperature sensing	Wearable smart devices	[23]
Capacitive mode sensors	CCNF, DES, NaOH, DA, PDA, AM	-	-	-	-	C = 0.99	Adhesion, frost resistance, moisture retention, mechanical properties	Monitoring human electrocardiogram and bioelectrode adhesion	[24]
	MoO_2_, a-CNTs, rGO, PVA	-	-	246.7%	-	-	Cycle stability, stretchability, high mechanical properties	Wearable portable electronic devices, self-healing energy storage devices	[25]
	AA, AM, CMC, ZnSO_4_	1.22 MPa	-	424%	2.9	C = 38	Mechanical performance, flexibility, tensile strain, sensing performance, electrochemical performance	Flexible energy storage and application in wearable electronic devices	[26]
	PAM, SA, HCl, ZnSO_4_	0.1654 MPa	−20 °C	408.2%	5.08	-	Mechanical properties (GF = 5.8), water retention, tensile sensing, adhesion, high sensitivity, and cycling stability	Detecting human movements, including fingers, arms, knees, ankles, and pulse	[27]
	PVA, AM, MBA, KPS, NMP	0.1622 MPa	−17.3 °C	826%	3.0	C = 21.7	High compressibility, frost resistance (−85 °C), high stretchability, self-adhesive elasticity, fatigue resistance	Distinguishing various human movements	[28]
Piezoelectric mode sensors	PVDF, AN, AAm, NaSS, APS	3.15 MPa	−20 °C	780%	-	-	High elongation, high toughness, frost resistance, self-powering performance, high sensitivity	Monitoring human movements, including finger and elbow bending, speaking, pulse, and gesture signal acquisition	[29]
	PVA, PSS, MXene, CNFs, Glycerol	-	−20 °C	15.3%	3.37	-	Frost resistance (−20 °C), tensile strength, piezoelectric mode activity, mechanical properties, moisturizing properties	Distinguishing between written letters and spoken words, and application in flexible wearable electronic products	[30]
	BNNT, PVDF, DMF, NMP	18.10 MPa	-	52.12%	-	-	Thermal conductivity, high waterproof and moisture-proof performance, mechanical strength, stretchability	Wearable sensing devices	[31]
	NaCl, CMC, BAPO-OH, Na-CMC	-	-	600%	2.27	-	Biocompatibility, stretchability, high sensitivity, repeatability	Joint movement, heart rate, vocal cord vibration, and surface muscle contraction	[32]
	BaTiO_3_, AM, DMAA, CQ/DPI, PEGDA	0.3 MPa	-	>100%	-	*p* > 1.5	Tensile performance (>100% strain), mechanical properties, sensing properties, high sensitivity	Monitoring human motions, including finger, wrist, and knee movements	[33]
	CS, APTES, EG, Gel/OCS-ABTO	0.01127 MPa	-	225%	2.18	-	High sensitivity, stretchability, biocompatibility, mechanical response characteristics, self-powering performance	Human motion gesture recognition, elbow flexion, knee flexion, and plantar pressure distribution	[34]
Battery-based sensing mode sensors	Ur, SA, ZIB, ZnCl_2_	-	-	-	-	-	Mechanical properties, self-healing properties, biocompatibility	Monitoring human motion and physiological signal	[35]
	HEA, AS, Gly, PTFE	0.16 MPa	−42 °C	3487%	1.65	B = 2.64	Stretching performance (160 kPa), self-healing (95%) performance, fatigue resistance, frost resistance	Monitoring various human movements and medical tests	[36]
	AAm, BR, DMSO, MBAA, Zn	-	−36.9 °C	-	-	B = 24	Fatigue resistance, frost resistance (−30 °C), stability, stretchability	Monitoring human movement at low temperatures	[37]
	NaCl, LiOH, ECH, K_4_Fe (CN)_6_·3H_2_O	-	-	-	-	B = 26.1	High mechanical properties, sensing performance, stretchability	Monitoring body temperature and human movement	[38]
	PAAM, CMC, TA, ZnSO_4_, AAM, PVDF	0.1063 MPa	−10 °C	469.2%	5.07	B = 0.76 ± 0.04	Stretchability (132 kPa), self-powered sensing performance, self-healing performance, self-adhesive performance	Monitoring various human movements	[39]

Note: The symbols marked with “-” in the table indicate that specific data was not reported in the original study. The appearance of these missing values is usually due to research focusing on other performance characteristics.

### 2.1. Conductivity

Hydrogels are unique conductive materials known for their inherent trace ionic conductivity [39]. However, this inherent conductivity is insufficient to meet the performance requirements for practical applications. To overcome this limitation, researchers have significantly enhanced their conductivity by integrating conductive materials into the structure and constructing cross-linked polymer networks. This approach introduces conductive materials to establish efficient electron transport pathways, while the cross-linked polymer network provides a stable framework to support these conductive materials. Conductive materials used in hydrogels are generally classified into two main types: ionic conductive materials and electronic conductive materials [40].

**Figure 1:**
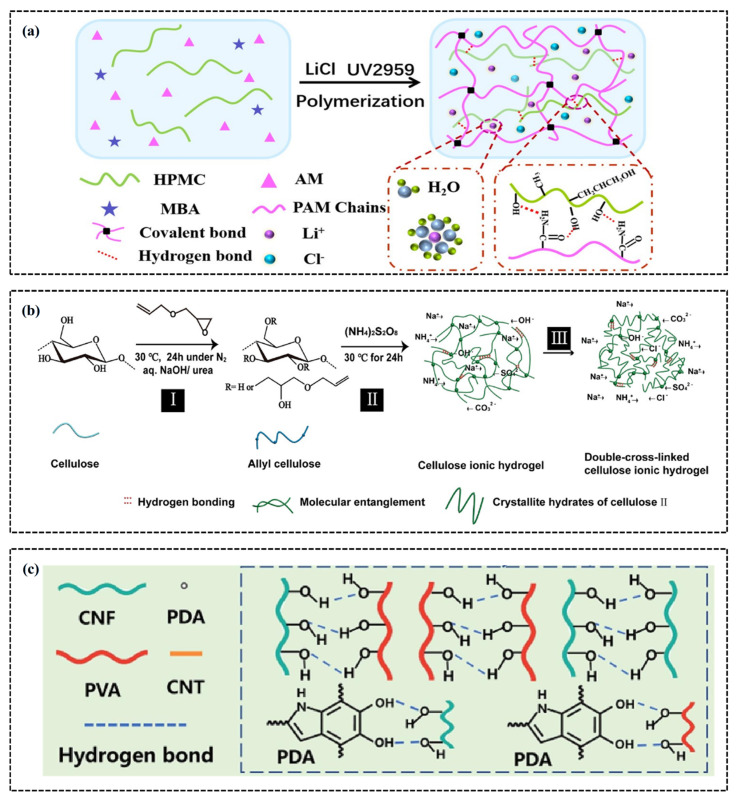
Conductivity of CH–FWSs. (**a**) Conductive ion flow diagram of PAM/HPMC/LiCl hydrogel [43]. (**b**) Schematic illustration of synthesis of DCIHs [44]. (**c**) The schematic preparation process of PVA/CNF@PDA/CNT hydrogel [49].

In ionic conductive hydrogels, the incorporation of free mobile ions, such as Fe^3+^, Ca^2+^, Li^+^, and anions like SO4^2−^ and Cl^−^, significantly enhances the conductivity of the hydrogel. However, high salt concentration can induce the salting out effect, referred to as the phenomenon where increased ionic strength in solution prompts strong interactions between ions and water molecules, leading to reduced solvation of polymer chains by water. This effect results in a relative increase in local polymer chain concentration, facilitates interchain interactions and aggregation, and consequently enhances hydrogel network formation and stability. Alternatively, various types of ionic polymers, including zwitterionic, anionic, and cationic polymers can be integrated, thereby providing new possibilities for developing high-performance flexible electronic devices [41,42]. For example, Qin et al. [43] developed a novel ionic conductive hydrogel by incorporating LiCl into a polyacrylamide/hydroxypropyl methylcellulose (PAM/HPMC) composite hydrogel. This hydrogel achieved a conductivity of 9.82 S/m, along with excellent adhesion and stability across a broad operating temperature range. Specifically, the introduction of conductive ions significantly improved the hydrogel’s tensile properties (1453%), tensile strength (135 kPa), and skin-like elasticity (9.18 kPa), making it suitable for the development of skin-like hydrogel sensors with high sensitivity (Figure 1a). Similarly, Tong et al. [44] employed a strategy involving the free radical polymerization of allyl cellulose with ammonium persulfate, g coupled with NaCl-induced physical crosslinking, to fabricate double crosslinked cellulose ionic hydrogels (DCIHs). These hydrogels demonstrate ~100% tensile strain at −24 °C and maintain high visual transparency in the low-temperature range of −30 to −16 °C. These findings show that DCIHs possess high stability, rapid response, and wide-ranging strain sensing capabilities, with a conductivity of 1.8 × 10^−5^ S/cm, excellent conductivity, and the ability to maintain stability at extreme temperatures, thereby offering new possibilities for the application of flexible electronic devices in diverse environments (Figure 1b).

Although ionic conductive hydrogels have significant advantages, their relatively low conductivity still limits their practical applications. To address this limitation, researchers have optimized the electronic conductivity of hydrogels by incorporating materials capable of releasing free electrons, such as carbon nanomaterials [45], graphene and its derivatives [46], conductive polymers [47]. For example, Peng et al. [48] synthesized a novel composite conductive hydrogel using self-reinforced cellulose, with lignosulfonic acid/single-walled carbon nanotube film/pore-reduced graphene oxide (Lig/SWCNT/HrGO) as a reinforcing agent. This hydrogel demonstrates exceptional tensile strength (112.3 MPa), area capacitance (1121 mF cm^−2^), and energy density (77.8 μWh cm^−2^). Notably, at a film electrode mass of 16.5 mg cm^−2^, it achieved an ultra-high area capacitance of 4110 mF cm^−2^ and an energy density of 285.4 μWh cm^−2^. In another study, researchers inspired by mussels developed a polyvinyl alcohol (PVA) hydrogel through in situ polymerization in PVA, polydopamine (PDA), and cellulose nanofiber (CNF) hydrogels/CNF@PDA/Carbon Nanotubes (CNT) composite conductive hydrogel [49]. PDA not only enhances the self-adhesive and self-healing properties of the hydrogel but also promotes the uniform dispersion of CNTs through noncovalent bonding. This created an efficient conductive network with a conductivity of 0.4 S/m, excellent mechanical properties, and antibacterial activity (Figure 1c).

### 2.2. Adhesiveness

**Figure 2:**
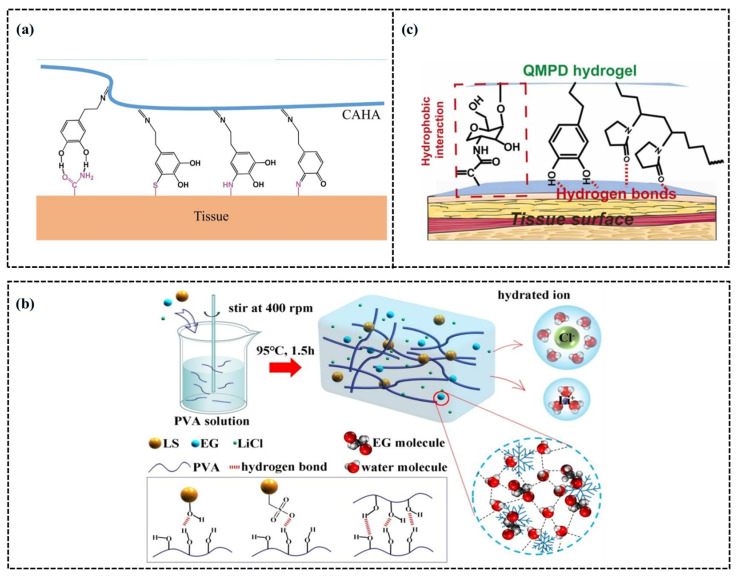
Adhesion of CH-FWSs. (**a**) Schematic illustration of a potential mechanism of the bioadhesion [52]. (**b**) Schematic diagram of PVA/LS/LiCl/EG hydrogel and the cross-linked structure [54]. (**c**) The illustration of hydrogel preparation from QCS-MA, PVP, and DA and its adhesion mechanisms to wet tissue surface [55].

In the field of human motion monitoring, sensor adhesion is a critical factor. Traditional sensors often rely on adhesive media like tape to attach to the skin. However, this adhesion may lose effectiveness due to movement or perspiration, thereby impacting the accuracy and reliability of monitoring data [50]. In contrast, hydrogels can utilize their inherent adhesiveness to directly and closely adhere to human skin without requiring additional adhesives [51]. They can be removed safely and non-invasively from the skin after use. This advantage has been widely acknowledged and applied in recent research on wearable electronic devices. Currently, hydrogel adhesion is achieved primarily through chemical and physical adhesion mechanisms [52]. Chemical adhesive hydrogels form covalent bonds or noncovalent interactions with tissue surfaces. For example, by incorporating dopamine (DA) into hyaluronic acid (HA)-based hydrogels, Ding et al. [52] significantly enhanced the adhesion of hydrogels to tissues and enhanced their rapid gelation and self-healing characteristics (Figure 2a). The study reported that the hydrogel’s adhesion strength increased to 90.0 ± 6.7 kPa after the introduction of DA. Additionally, dopamine quinone, an oxidative derivative of DA, forms stable covalent bonds with amino groups on the tissue surface through the Schiff base reaction, further improving adhesion performance to 384.6 ± 26.0 J m^−2^, surpassing both unmodified HA hydrogels and commercial fibrin adhesives.

Physical adhesion hydrogels achieve high adhesion efficiency by altering the internal network structure. This approach increases the contact surface area with biological tissue, either by incorporating microstructures into the hydrogel or modifying its porosity [53]. This design strategy not only enhances the adhesion of hydrogels but also modifies their mechanical properties, including elastic modulus and toughness, enabling them to adapt to the changing organizational environment and achieve stronger adhesion. For instance, Yang et al. [54] employed PVA as a matrix and formed hydrogen bonds via a one-pot method utilizing its hydroxyl groups, as depicted in Figure 2b. This approach confers self-healing properties on the hydrogel, improves its multi-matrix adhesion, and broadens its application in motion sensors. In another study, researchers utilized polyacrylic acid (PAA), chitosan (CS), and polydopamine (PDA) to develop a solid double-network (DN) hydrogel via physical and chemical cross-linking, as illustrated in Figure 2c [55]. Introducing Al^3+^ ions as the “bridge” connecting components and coordination bonds further enhanced the hydrogel’s porous network, thereby improving its adhesion. The obtained PAAc/PDA/CS (ADC) hydrogel exhibits excellent mechanical properties and high sensitivity, with an adhesion strength on pig skin reaching up to 85.2 kPa.

### 2.3. Self-Healing Ability

The high plasticity and self-healing abilities of hydrogels have attracted significant attention in the field of wearable strain sensors. The self-healing capability of sensors is crucial for monitoring performance, as it allows for recovery after damage, ensuring stability and prolonging their lifespan [56]. When the sensor is damaged, the hydrogel can facilitate the recovery of its performance through its inherent repair mechanism or external stimulation, owing to the high mobility of polymer chains and the low rigidity of the structure. External stimulation including self-healing agents, heat, moisture, ultraviolet radiation, and electric energy can trigger dynamic crosslinking reactions in the hydrogel [57].

**Figure 3:**
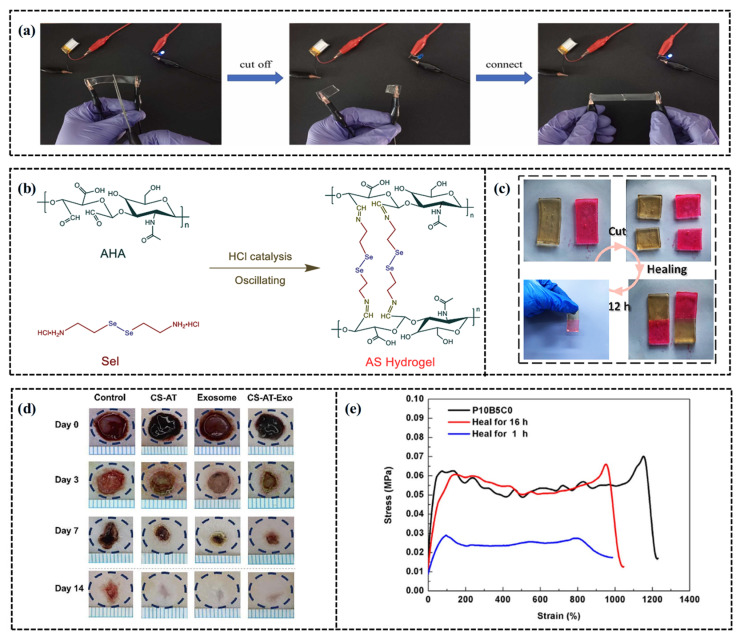
Self-healing of CH-FWSs. (**a**) The brightness of LED lamp after cutting PEG-HEMA hydrogel at 25 °C and self-healing was compared [58]. (**b**) Chemical structural formulae for the synthesis of AS hydrogels [61]. (**c**) The demonstration of self-healing process [62]. (**d**) Representative images of diabetic wound healing in rats treated with PBS (control), CS-AT hydrogels, exosomes, and CS-AT-Exo hydrogels [68]. (**e**) Stress–strain curves of original and self–healing PVA–Borax hydrogel specimens denoted as P10B5C0 (mass ratio of 10:5:0) at various healing times, respectively [69].

For example, researchers developed a conductive double-network polyethylene glycol-hydroxyethyl methacrylate (PEG-HEMA) hydrogel with antifreeze properties, UV resistance, and self-healing capabilities by utilizing PEG and water as the mixed solvent and HEMA as the monomer. The self-healing capability of the hydrogel is activated by external stimulation from the mixed solvent as shown in Figure 3a [58]. In contrast, without external stimulation, the self-healing efficiency of hydrogels is significantly reduced. While external stimulation can enhance the self-healing performance of hydrogels, the lack of dynamic physical bonds in the hydrogel network limits its natural diffusion capability at the cutting interface. However, the self-repair mechanism of hydrogels effectively over-come this limitation by utilizing their internal dynamic cross-linking networks, which include covalent bonds, noncovalent bonds, and multiple dynamic interactions, enabling self-repair without external stimuli [59,60]. For example, Jia et al. [61] developed an ultra-fast, low-temperature self-healing ionic hydrogel (ASCL) based on aldehyde hyaluronic acid (AHA), utilizing the synergistic effects of dynamic Schiff base bonds and disulfide bonds, as shown in Figure 3b. This hydrogel forms a 3D network structure that provides the foundation for rapid self-healing capability (achieving rapid self-healing within 5 min at a low temperature of −20 °C) through these reversible dynamic bonds. Additionally, Liu et al. [62] developed a novel double-network hydrogel by dynamically coordinating Tara tannin with Fe^3+^ and incorporating the CSMA modifier, as shown in Figure 3c. This design takes advantage of the dynamic coordination between Tara tannin and Fe^3+^, as well as hydrogen bonding involving phenolic hydroxyl and carboxyl groups, while the addition of CSMA increases the cross-linking density of the network. These dynamic bonds can be quickly reorganized when the hydrogel is damaged, promoting rapid self-healing and improving both the mechanical strength and self-healing efficiency of the hydrogel.

Supramolecular interactions stem from noncovalent forces between molecules. Although these interactions are weak, their collective presence significantly influences macroscopic properties. These interactions provide dynamism and reversibility to intermolecular binding, enabling rapid self-repair of materials after damage [63,64,65]. Dynamic covalent bonds, such as Schiff base (imine), acyl hydrazone, and boronic ester bonds, are integral to supramolecular systems due to their ability to reversibly break and reform under specific conditions like changes in pH or temperature [66,67]. The Schiff base reaction, a reversible chemical process, forms imine bonds through interactions between amino groups and aldehydes or ketones, conferring self-healing properties to hydrogels. For example, Wang et al. [68] developed a fast self-healing conductive hydrogel utilizing the Schiff base reaction between the amine group of chitosan grafted with an aniline tetramer (CS-AT) and the aldehyde group of polyethylene glycol (PEG-DA) capped with benzaldehyde, as shown in Figure 3d. This hydrogel demonstrated self-healing within three hours at 37 °C, with the aniline tetramer significantly enhancing its performance. Similarly, acyl hydrazone bonds, formed via reactions between carboxylic acid and hydrazine, undergo reversible fracture and recombination in response to pH or temperature changes, granting rapid self-healing capabilities to hydrogels. Meanwhile, borate ester bonds, formed through interactions between diol and boric acid, particularly the cyclic borate ester (NCB) structure combined with internal B-N coordination, enhance hydrogel stability against water and heat while maintaining self-healing properties. For example, researchers have synthesized a new type of hydrogel by crosslinking PVA with borax and incorporating polypyrrole nanotubes, as shown in Figure 3e [69]. The B(OH)_3_ and B(OH)_4_ produced from the borax decomposition react with hydroxyl groups in PVA, rapidly forming reversible boronic ester bonds. This dynamic chemical crosslinking not only significantly improves the mechanical strength of the hydrogel (up to 0.72 MPa) but also achieves 83% self-repair efficiency and rapid repair capability (15 s) (Figure 3d).

### 2.4. Biocompatibility

**Figure 4:**
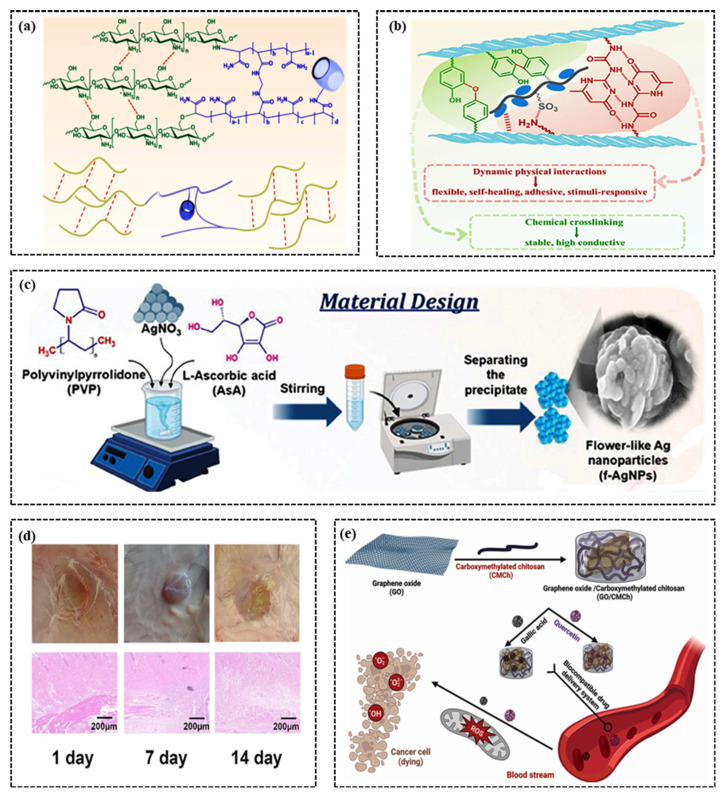
Biocompatibility of CH-FWSs. (**a**) Schematic of the synthesis of CxPy hydrogel and interactions between polymer networks and water-soluble polypyrrole [71]. (**b**) Schematic of the preparation of GUT-PP hydrogel based on GUT and PEDOT:PSS-Tyr by combining chemical cross-linking and stimuli-responsive physical interactions [72]. (**c**) Preparation process of multifunctional PKB3/F-AgNPs hydrogel materials [73]. (**d**) Changes in biocompatibility of hydrogels during 1–14 days [74]. (**e**) Preparation process of graphene oxide/carboxymethyl chitosan (GO/CMCh) nanocomposite hydrogel [75].

The bio-compatibility of hydrogels is influenced by various factors, including chemical composition, molecular structure, surface characteristics, and pore structure, which collectively determine their ability to interact with biological tissues [70]. For example, researchers have designed an innovative conductive hydrogel using water-soluble polypyrrole (PPy) and chitosan, as shown in Figure 4a. This hydrogel leverages the biocompatibility of chitosan and the ionic structure of PPy to achieve excellent biocompatibility [71]. MTT assays and fluorescence microscopy demonstrated a high cell survival rate and normal proliferation of the hydrogel on NCM460 and NIH3T3 cells, even under high-concentration conditions. In another study, Sun et al. [72] constructed a biocompatible multifunctional hydrogel through chemical crosslinking and stimulus-responsive physical interactions, as shown in Figure 4b. By functionalizing gelatin with Upy/Tyr, the hydrogel exhibited robust dynamic physical interactions and stable covalent crosslinking, resulting in exception-al biocompatibility. Notably, this hydrogel did not cause skin allergic reactions during prolonged use in human health monitoring.

At present, different mechanisms and materials are combined in CH-FWSs to improve biocompatibility. Cheng et al. [73] developed a novel hydrogel composed of PVA, konjac glucomannan (KGM), borax (B), and flower-shaped silver nanoparticles (F-AgNPs), as shown in Figure 4c. This hydrogel features a layered network structure that not only enhances tensile strength and self-healing performance but also demonstrates excellent and stable biocompatibility. It presents a cost-effective solution for detecting bodily movements and facial expressions. In another study, a multifunctional hydrogel was created using amylopectin, PVA, lithium chloride, and glycerin. This hydrogel forms hydrogen bonds between amylopectin and PVA, with glycerin and lithium chloride improving its mechanical properties and frost resistance [74]. The study found the hydrogel to be biocompatible in mice, without causing allergic reactions, as shown in Figure 4d. To further enhance the biocompatibility of hydrogels, researchers are investigating various reinforcement materials and advanced preparation technologies. For instance, Ahmed M et al. [75] developed a nanocomposite hydrogel incorporating graphene oxide/carboxymethyl chitosan (GO/CMCh) to regulate the release of polyphenol antioxidants (Figure 4e). This nanocomposite hydrogel demonstrated an extremely low hemolysis rate (<1%) and excellent biocompatibility, confirming its potential for clinical use.

In conductive hydrogel preparation, potential toxicity risks primarily arise from residual chemicals and solvents. During synthesis, low-toxicity or non-toxic solvents should be selected whenever possible. Residual toxic solvents can be minimized through optimized preparation and post-treatment methods, thereby enhancing the biocompatibility and safety of conductive hydrogels. In recent years, numerous conductive hydrogels based on aqueous hydrogels and ionic gels have been reported, using water and ionic liquids as solvents, respectively. However, these hydrogels exhibit ionic liquid leakage issues, particularly under compressive and tensile deformation. Their toxicity impedes practical applications in wearable electronics and restricts the long-term stability of gel-based electronic devices. Consequently, developing stretchable conductive hydrogels with stability and biosafety could pioneer new directions for flexible electronics. Liu’s team [76] introduced supramolecular encapsulation, utilizing biocompatible PVA to encapsulate non-toxic, ionizable natural molecules (IP6) via high-density hydrogen bonding. This approach yielded a solvent-free supramolecular ion conductive elastomer (SF supra ICE) that integrates stability and biosafety. Unlike hydrogels and ionic gels, SF supra ICE contains no solvents. This solvent-free characteristic provides long-term stable skin-like mechanical properties and electrical conductivity (room temperature ionic conductivity up to 3.3 × 10^−2^ S m^−1^) while ensuring excellent biosafety. Additionally, the resulting multifunctional skin electronics enable human electrophysiological signal acquisition/monitoring, human–computer interaction, and precise diagnosis/treatment, showing broad application potential in flexible electronics. Additionally, Zhao’s team [77] employed ethylene glycol (EG) as a solvent (instead of water) to develop a non-toxic, high-conductivity, low-stiffness, high-tensile, and self-healing composite conductive hydrogel (Ag LM PVA). EG exhibits a low evaporation rate under ambient conditions, preventing rapid hydrogel dehydration and thereby enhancing stability and biocompatibility. They utilized Ag LM PVA as EMG sensing electrodes. Its self-healing properties enable manual reconfiguration for capturing physiological signals from various body positions. Additionally, the gel’s characteristics facilitate the construction of personalized electrodes for addressing complex clinical challenges. These results demonstrate the strong application potential of Ag-LM-PVA organic gels in soft robotics, flexible electronics, and medical devices. In another study, Sun et al. [78] developed a deep eutectic solvent (DES)-based eutectoid gel. This gel exhibits high ionic conductivity, low vapor pressure, excellent thermal stability, and non-toxicity, addressing the high toxicity and cost issues of traditional ionic liquid (IL)-based gels. Lei et al. [79] developed single-network and multi-network hydrogels using water-soluble N-type cationic semiconductor polymers. They employed disodium 1,3-benzenedisulfonate (DBS) as a crosslinking agent to form N-type semiconductor hydrogels via anionic electrostatic crosslinking. The P(PyV) hydrogel [P(PyV)-H] film was fabricated by spin-coating P(PyV) solution onto the substrate followed by immersion in a DBS DMSO solution. These hydrogels display unique N-type semiconductor properties and can be used to construct logic circuits and amplifiers. The authors also confirmed that these semiconductor hydrogels exhibit low cytotoxicity and excellent biocompatibility, enabling simultaneous capture and amplification of electrophysiological signals.

### 2.5. Antifreezing and Antiswelling Properties

The anti-freezing and anti-swelling properties of conductive hydrogels significantly impact their applications in wearable sensing under extreme conditions (e.g., low temperatures and sweat immersion). The hydrogel’s three-dimensional polymer framework typically contains hydrophilic segments. These segments will freeze under sub-zero temperatures, slowing carrier transport and causing significant reductions in flexibility and conductivity [80]. Additionally, in aqueous environments, significant water infiltration into the hydrogel’s network structure causes swelling. While hydrogel swelling presents a double-edged sword, it benefits biomedical applications like tissue engineering, drug delivery, and controlled release [81].

However, for electronic applications, hydrogel swelling represents an inherent drawback. Swelling induces polymer chain dilution, reducing mechanical properties while potentially affecting conductivity and sensor stability. Researchers have explored natural materials, innovative fabrication techniques, and diverse structural designs to optimize conductive hydrogels’ anti-freezing and anti-swelling properties while ensuring long-term stability in complex environments. For instance, Gao et al. [82] developed a conductive hydrogel composite system featuring diverse hydrogen bond interactions. By incorporating the natural biomolecule lignin (AL), they significantly enhanced the hydrogel’s frost resistance (−25.88 °C) and swelling resistance (optimal swelling rate of 10%). Results demonstrated that the conductive hydrogel maintained mechanical properties (elongation at break > 300%, tensile strength > 0.13 MPa) and electrical conductivity (0.18 S/m) after prolonged water immersion. The resulting flexible sensors accurately detect human and aquatic organism movements in water. The wireless sensor system transmits data via Bluetooth and displays EMG signals comparable to commercial Ag/AgCl gel electrodes. This green, low-cost, sustainable anti-freezing and anti-swelling conductive hydrogel shows significant potential for low-temperature and underwater sensing applications. In another study, Feng’s team [83] employed a “molecular locking hydrophobic shield” dual strategy. They formed hydrophobic regions via room-temperature methylacrylamide self-assembly and enhanced gel-water molecule interactions to achieve simultaneous anti-freezing and anti-swelling properties (7-day swelling rate of 11%). Hydroxyl groups of acrylamide and cellulose nanofibers, along with methylacrylamide amide groups, form multiple dynamic hydrogen bonds. This enables the hydrogel to achieve a tensile strength of 554 kPa and rapid self-healing (92.5% efficiency within 3 h). The ionic liquid serves as both a conductive pathway and “water-locking agent,” enabling stable strain signal output across the extreme temperature range of −50 °C to 80 °C. The resulting conductive hydrogel can monitor human movement across wide temperature ranges, offering a novel solution for flexible sensing materials.

### 2.6. Degradability and Sustainability

Society requires electronic materials that meet sustainability standards to facilitate the development of easily disposable green devices. Although conductive hydrogel-based flexible electronic materials offer low cost and high performance, sustainability remains a primary challenge. Developing environmentally friendly, biodegradable alternative materials is essential. As research into the environmental impact of electronic waste increases, there is a growing emphasis on finding environmentally friendly, biodegradable materials and “Eco design” solutions. This ensures conductive hydrogels maintain high sensitivity in wearable devices while achieving complete biodegradability at the end of their life cycle. For instance, Aljarid et al. [84] developed a biodegradable, highly sensitive piezoresistive graphene hydrogel sensor by combining natural materials (algae, rock salt, and water) with graphene. This novel sensor offers an environmentally friendly wearable solution without compromising sensing performance. It can detect masses as low as 2 mg (equivalent to a raindrop impact), demonstrating superior sensitivity and accuracy compared to conventional polymer-based wearable devices. In another study, Chen’s team [85] proposed an innovative strategy to develop an “all-natural antifreeze conductive hydrogel” by leveraging the strong coordination between natural polymers (cellulose) and natural minerals (bentonite). The developed hydrogel demonstrates high strength (compressive strength up to 3.2 MPa) and high toughness (fracture energy up to 0.76 MJ m^−3^). Additionally, the intercalation structure formed by cellulose molecules and nano-bentonite creates a nanoconfinement effect. This provides a high-speed migration pathway for conductive ions within the hydrogel, thereby significantly enhancing its conductivity. The hydrogel’s ionic conductivity in water at 25 °C and −20 °C reached 89.9 mS cm^−1^ and 25.8 mS cm^−1^, respectively. As a biosensor, the cellulose hydrogel enables stable monitoring of human movement and physiological signals, showing great application potential in flexible wearables. This study offers new insights into the sustainable preparation of biomass-based hydrogels. Subsequently, Chen et al. [86] reported a cellulose-based stiffness-adjustable intelligent material inspired by the sea cucumber’s dermal structure. They utilized the differential responsiveness of cellulose molecules (Cel) and polyacrylamide molecules (PAAm) to ethanol stimulation, constructing a dynamically reconfigurable supramolecular network. This resulted in an intelligent material (Cel-PAAm) with dynamically switchable mechanical properties, exhibiting exceptional soft-hard transition performance. Additionally, the material possesses unique processability, recyclability, and self-healing capabilities akin to living organisms. This makes it promising for applications in intelligent robotics, implantable medical electronics, additive manufacturing, and smart protective systems.

## 3. Conductivity Mechanism of CH-FWSs

**Figure 5:**
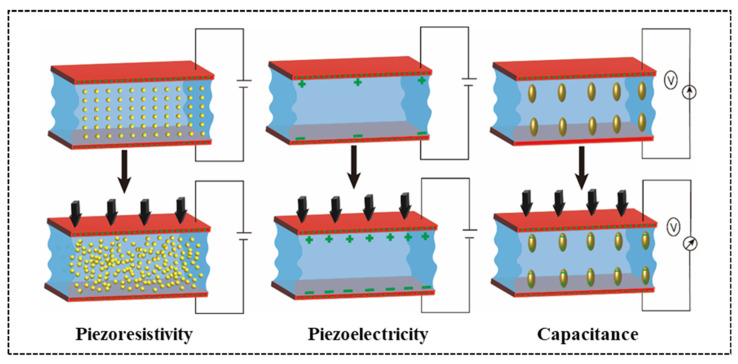
Conductive mechanism based on CH-FWSs, including resistance, capacitance and piezoelectric mode.

CH FWSs play a pivotal role in motion-related applications attributable to their distinctive conductive sensing mechanisms. Researchers have developed various conductive modes suitable for different application scenarios by ingeniously constructing conductive network structures and continuously innovating materials to meet diverse motion monitoring needs. The common resistance, capacitance, and piezoelectric modes, which rely on distinct physical mechanisms, provide enormous potential for optimizing the performance and expanding the applications of CH FWSs, as shown in Figure 5.

### 3.1. Resistance Mode

Resistance mode CH-FWSs are extensively used to detect small deformations and stresses due to their remarkable sensitivity. These sensors function by measuring the geometric deformation in a structure under applied force, which results in variations in resistance [87,88,89,90]. When employed in flexible wearable sensors, hydrogels primarily motor the deformation caused by external forces, like stretching or extrusion, which induce resistance changes. In practical applications, conductive hydrogels operate via either ionic or electronic conduction. Regardless of the conduction type, the sensing mechanism relies on the influence of external deformation on resistance [91]. This detection mechanism based on resistance change makes hydrogels particularly advantageous for dynamic monitoring of human movement or mechanical deformation.

**Figure 6:**
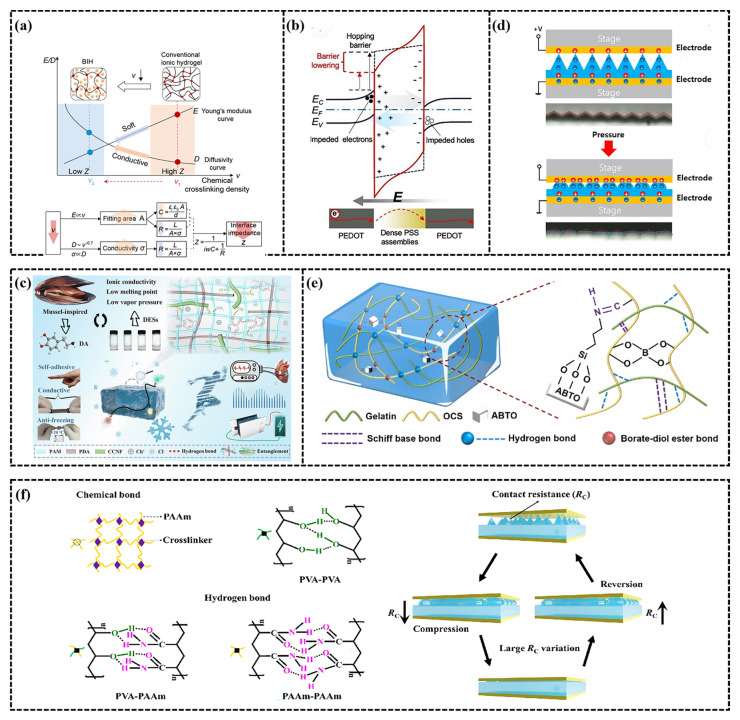
Resistance mode mechanism of CH FWSs. (**a**) The relationship between chemical crosslinking density, Young’s modulus and ion diffusion characteristics in covalently crosslinked ionic hydrogels. The lower cross-linking density in the hydrogel polymer chain enhances the interface performance by reducing the contact impedance (Z), increasing the tissue fitting area (A) and increasing the ionic conductivity (σ) [94]. (**b**) The mechanism of enhanced electron transport in the hydrogel. Under electric field induced local polarization, dense PSS components with ultra-high ion concentration significantly reduce the electron hopping barrier between PEDOT microcrystals. The gradient color of dense PSS components represents polarization under an applied electric field, where darker shadows indicate higher PSS concentration and more negative charges, while lighter shadows indicate more positive charges [99]. (**c**) Internal network structure of DES/CCNF hydrogel and interaction between components [103]. (**d**) Schematic diagram of EDL formed when pressure is applied to the hydrogel-based pressure sensor [93]. (**e**) Molecular interaction between gelatin, OCS and ABTO in the hydrogel [32]. (**f**) Covalent bonds between PAAm chains, rich inter chain hydrogen bonds between PVA-PVA, PVA-PAAm and PAAm PAam chains, and resistance signal change mechanism of pyramid microarray hydrogel-based sensors for external pressure [110].

The resistance (R) of ionic conductive hydrogels, a specialized type of resistor, follows Ohm’s law and is governed by the resistivity (ρ), the length (L) of the conductive material, and the cross-sectional area (S), expressed as R = ρL/S [92]. This implies that the resistance of the hydrogel increases with an increase in its length or a decrease in cross-sectional area. Consequently, when the hydrogel undergoes stretching or compression during motion monitoring, causing changes in its dimensions, its resistance changes accordingly. This change in resistance is detected by the ionic conductive hydrogel sensor and converted into electrical signals to monitor small deformations, such as muscle contraction and joint activity [93]. Wang et al. [94] developed an innovative resistive wearable ionic conductive hydrogel sensor using 2-acrylamide-2-methylpropane sulfonic acid (AMPS) polyelectrolyte ions, as depicted in Figure 6a. This design not only endows the hydrogel with superior mechanical properties and conductivity (8.55 S m^−1^) but also improves its adaptability to soft tissue by reducing contact impedance and enhancing signal fidelity. This makes it suitable for applications such as EMG monitoring. According to Ohm’s law, the resistance of electronic hydrogel sensors depends on a combination of factors including resistivity (ρ), length (L), and cross-sectional area (S) [95]. The electronic conductive hydrogels exhibit distinct contact resistance and electronic tunneling effects [96,97]. At lower concentrations of conductive particles, the tunneling effect facilitates efficient charge transfer, creating a low-resistance electronic pathway. As the concentration of conductive particles increases and surpasses the percolation threshold, a continuous conductive network forms, driven by the contact resistance effect. This network significantly reduces resistance and enhances electronic conductivity, rendering the sensor highly sensitive and stable under both small and significant strains [98]. Despite these advancements, resistive wearable sensors based on conductive hydrogels still have room for improvement in both conductivity and sensitivity. To address these challenges, researchers have explored various methods to enhance the situation. For instance, Li et al. [99] fabricated a highly conductive electronic hydrogel using PEDOT: PSS, as depicted in Figure 6b. The PSS component of the hydrogel substantially decreases the energy barrier for electron hopping between PEDOT microcrystals, thereby enhancing electron transmission. The charge transfer rate reaches up to 2 × 10^6^ m/s^−1^, which is approximately 5 orders of magnitude higher than that based on ion transmission. This offers a novel approach to enhancing the sensitivity and conductivity of resistive wearable sensors based on conductive hydrogels.

### 3.2. Capacitive Mode

Capacitive mode CH-FWSs detect physical deformation by monitoring changes in capacitance. Compared to resistive sensors, they offer notable benefits, including reduced measurement latency and enhanced response speed, while maintaining high linearity with the measured physical quantity [100]. Typically, these sensors consist of a dielectric layer sandwiched between two conformal electrodes, forming a parallel plate capacitor. The capacitance C is determined by the formula C = ϵS/d, where ϵ is the dielectric constant, S is the electrode area, and d is the electrode spacing [101]. When subjected to strain or pressure, the dielectric layer’s geometry changes, resulting in a variation in capacitance. The material composition and structure of such capacitive mode CH FWSs significantly influence their performance, since they enhance measurement sensitivity and improve response speed [102].

Recent research highlights significant achievements in Capacitive mode CH-FWSs, driven by innovative materials and microstructure design. For instance, Li et al. [103] developed a multifunctional conductive hydrogel for a novel dopamine supercapacitor by employing cellulose carboxylate nanofibers (CCNF), eutectic solvents (DES), and alkaline solutions as polymerization media. The combination of this hydrogel DES and alkaline solution replaced pure DES to offer conditions for oxidative electrolyte polymerization, as illustrated in Figure 6c. Furthermore, the design of microstructures can substantially enhance the performance and conductivity of capacitive strain sensors [104]. For example, Byeong et al. [105] developed a pyramid-structured hydrogel via the soft lithography process, as shown in Figure 6d. In their research, they minimized the amount of crosslinking agent to enhance the sensitivity of the pyramid structure, enabling the hydrogel to detect both low pressure and high pressure (2000 kPa). These designs enhance capacitance change efficiency by increasing the sensor’s surface area and optimize performance by improving linearity and measurement accuracy.

### 3.3. Piezoelectric Mode

Piezoelectric mode CH-FWSs are crucial components in monitoring and detection systems owing to their self-powered characteristics and exceptional sensitivity [106]. In the design of piezoelectric CH-FWSs, the hydrogel is typically positioned between two electrodes, leveraging its piezoelectric properties to generate charges under mechanical strain, thus enabling the conversion of electrical signals. The efficiency and magnitude of this conversion are directly influenced by the direction of strain and the measurement method [107]. This sensor is typically fabricated from piezoelectric materials and other materials that generate charges in response to mechanical strain, thereby achieving a self-powering effect [108]. Recent advancements have focused on the use of piezoelectric mode CH-FWSs in energy harvesting by integrating piezoelectric materials into devices to convert mechanical energy into electrical energy, thus achieving high-power output [109].

Despite their significant potential in self-powered electronic devices, piezoelectric sensors face challenges, such as insufficient biocompatibility and mechanical flexibility, particularly for applications in health monitoring or as flexible devices, limiting their widespread use in practical scenarios. To address this challenge, Fu et al. [32] developed a high-performance, self-powered voltage hydrogel sensor through an innovative combination of PVDF with durable polyacrylonitrile (PAN) hydrogels, as shown in Figure 6e. This sensor utilizes the high dipole arrangement of the β-phase of PVDF, exhibiting an excellent piezoelectric response. The interaction between PVDF and PAN promotes the formation of a three-phase PVDF, significantly boosting its piezoelectric properties and achieving a piezoelectric coefficient of up to 30 pC/N. The sensor generates an electrical output of 30 mV and a current of 2.8 pA, with a response time of merely 31 milliseconds. This remarkable piezoelectric performance enables it to accurately detect physiological movements, such as finger bending, pulse, and vocal cord vibrations. Additionally, its self-powered nature reduces dependence on external power sources, enhancing its convenience and usability. Zhang et al. [110] prepared a pyramid microarray piezoresistive pressure sensor using polyacrylamide (PAAm)/polyvinyl alcohol (PVA), as shown in Figure 6f. This pyramid microarray modified ionic conductive PAAm/PVA hydrogel with mechanical properties can produce large contact resistance changes in response to external pressure, reaching a maximum sensitivity of 2.27 kPa and a detection limit of 9.0 Pa.

### 3.4. Triboelectric Mode

Frictional electric mode sensors have become an important development direction in the field of flexible wearable sensors in recent years. Frictional electric sensors have the advantages of high sensitivity, fast response, and no need for external power supply. Its basic working principle is based on the coupling of frictional electrification and electrostatic induction [111]. When two different materials come into contact and separate, due to the different charge distributions on the surface of the materials, charge transfer occurs at the contact surface, resulting in a potential difference between the materials. This potential difference can drive electrons to flow in the circuit, thereby generating current. Frictional electric sensors are usually composed of a friction layer, an electrode layer, and a substrate, and the material selection of the friction layer is crucial to the performance of the sensor [112]. Common friction layer materials include polytetrafluoroethylene (PTFE) [113], polydimethylsiloxane (PDMS) [114], polystyrene (PS) [115], etc. In recent years, researchers have developed a series of high-performance triboelectric sensors by combining the conductive hydrogel with the triboelectric effect. For example, Xie et al. [116] developed a new type of hydrogel triboelectric nano generator (TENG) based on the different electronic affinities between PTFE and PAHMC hydrogels for human motion monitoring. They proved that when the positive and negative triboelectric layers are in contact, electrons are transferred from PAHMC hydrogel to PTFE layer, and positive charges remain in PAHMC hydrogel layer; When separated, due to electrostatic induction, the two electrodes generate opposite charges, creating a potential difference that drives electrons to flow from the electrodes to the ground. In addition, He et al. [117] developed a sodium polyacrylate (SP)/polyvinyl alcohol (PVA) hydrogel friction nano generator (SP-TENG) using PDMS as the friction layer, which is used for the stretchable friction nano generator, proving its potential for energy collection and basketball sports monitoring.

The stable and durable continuous power generation of frictional electric mode sensors in extreme and complex environments remains a huge challenge. In order to solve this challenge, Jiang et al. [118] confirmed that the strong hydrogen bond with water molecules significantly enhanced the freezing resistance (−80 °C) and drying resistance of the gel by introducing glycerol into the hydrogel friction nano generator material. This improvement enabled the hydrogel sensor to maintain stable performance in long-term use. In addition, the design of frictional electric sensors in the field of motion monitoring also needs to consider their adhesion and comfort with human skin. For example, Yu et al. [119] developed a friction nano generator based on polyvinyl alcohol/tannic acid hydrogel. This hydrogel has high tensile, conductivity and adhesiveness, can closely fit human skin, and is suitable for health monitoring under various sports conditions.

## 4. Modification Strategies of CH-FWSs

**Figure 7:**
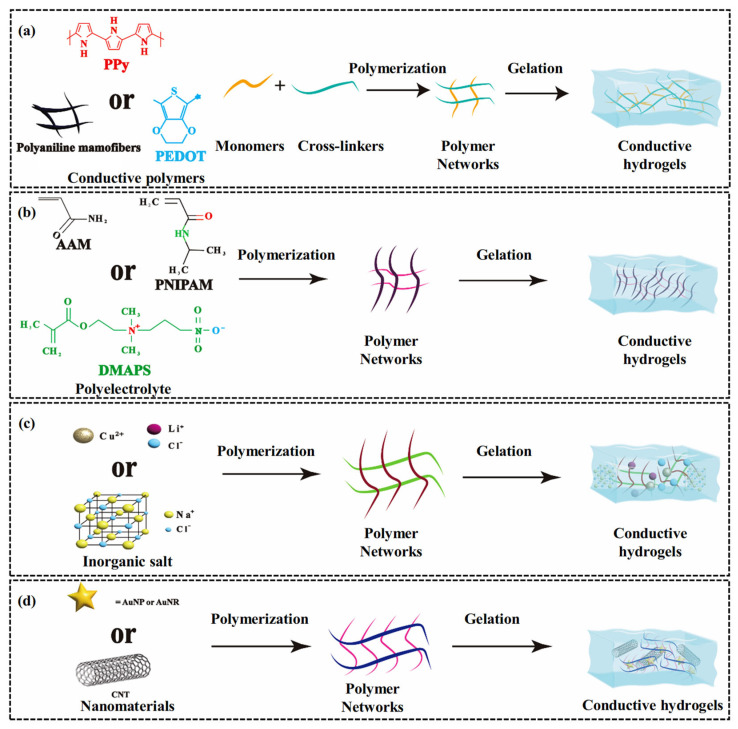
Shows the schematic diagram of conductive hydrogel modified by polymer (**a**), polyelectrolyte (**b**), inorganic salt (**c**) and nano material (**d**).

In the flexible electronics field, CH FWSs have been significantly enhanced through diverse modification techniques. These techniques impart adaptability to extreme environments, excellent sensitivity, and broad detection capabilities, thereby driving innovation in wearable flexible electronic devices. This chapter categorizes and describes CH FWSs according to distinct modification strategies. These include introducing conductive polymers, adding polyelectrolytes, doping with inorganic salts, and applying nanomaterials, as illustrated in Figure 7. These strategies enhance sensor performance across multiple dimensions and broaden its application scope.

### 4.1. Bonding of Conductive Polymers

**Figure 8:**
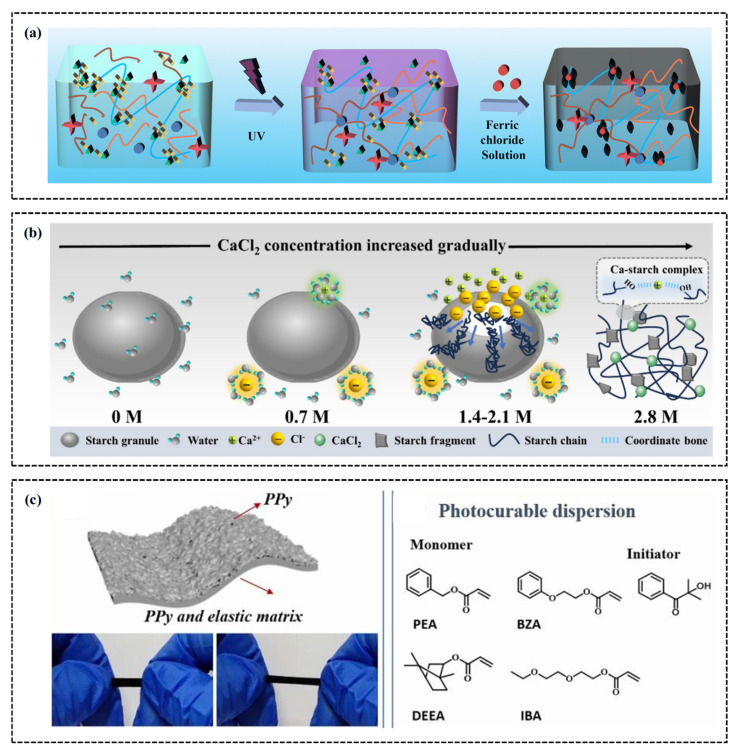
Capacitive mode mechanism of CH FWSs. (**a**) Schematic illustration of the fabrication process and properties of the TN hydrogel [125]. (**b**) Schematic diagram representing the structural disorganization of starch by CaCl_2_ solutions [126]. (**c**) Schematic illustration and photographs of PPA-2% for example. and chemical structures of four acrylate monomers (PEA, DEEA, BZA, IBA) and initiator (Darocur 1173) used to prepare PPAs [127].

The basic principle of conductive hydrogel composed of polymer (CP) is that the conjugated structure in the conductive polymer chain segment can promote the rapid transfer of electrons to form an efficient charge transfer channel [120]. CP-based hydrogels are classified into natural and chemical polymers. The natural polymer category primarily comprises polysaccharides (cellulose alginate, hyaluronic acid, and chitosan), peptides (gelatin, silk fibroin), and other natural polymers (starch, guar gum, agarose, and glucan) [121]. Chemical polymers mainly include polyacrylic acid (PAA), poly(methyl methacrylate) (PMA), polyacrylamide (PAM), polyaniline (PANi), and poly(3,4-ethylenedioxythiophene)/polyphenylene sulfide (PEDOT) [122,123,124]. A key molecular structural feature of these CPs is the presence of alternating single and double covalent bonds, which contributes to their high reversibility and stability in electrochemical processes. By adjusting the molecular structures of the dopant and CP, other properties of the conductive polymer hydrogel sensor can be significantly enhanced. For example, Hu et al. [125] developed a three-network hydrogel sensor by incorporating agarose (AG)/SBMA into PPy (Figure 8a). The sensor’s design involves the conjugated π system formed by the alternating single and double bonds of PPy in the hydrogel, the double helix beam cross-linking created by hydrogen bonding in the linear molecules of AG, and the interactions between sulfonic groups and quaternary ammonium cations, resulting in excellent mechanical and electrical properties (0.514 S/m^−2^).

Furthermore, optimizing the microstructure of CPs can significantly enhance the conductivity of conductive polymer hydrogels. For example, Xu et al. [126] demonstrated the versatility of starch hydrogels through polymer amorphization. In their study, calcium chloride-induced inhibition effectively suppressed starch retrogradation, thereby inducing the formation of uniform polymer chain aggregates, achieving adjustable polymer amorphization and coexistence of free and hydrogen-bonded hydroxyl groups, as shown in Figure 8b. This multi-scale microstructure endows the starch-based hydrogel with excellent properties, such as good water retention, high transparency (86.39%), improved self-adhesion, and self-repair capability. In addition, researchers incorporated PPy nanofillers with acrylate (MA) and polyacrylate (PMA), using PMA as the matrix to produce a conductive polymer hydrogel sensor with outstanding tensile properties, conductivity, sensitivity, and fatigue resistance, as shown in Figure 8c [127]. This sensor demonstrates superior light absorption (>98%) across a wide spectral range (250–2400 nm), presenting significant potential for applications in human motion monitoring and wearable thermal therapy devices. A notable advantage of this method is the concentration gradient filling of PPy nanowires, which significantly enhances the strain-sensing capability and the photothermal conversion efficiency of the sensor.

### 4.2. Combination of Polyelectrolytes

**Figure 9:**
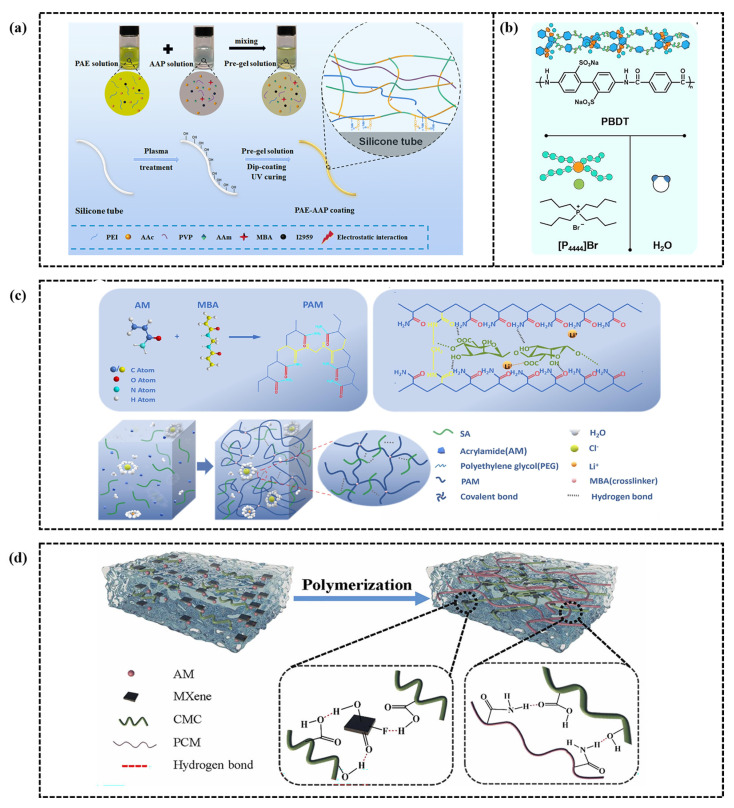
Piezoelectric mode mechanism of CH FWSs. (**a**) Preparation of pre-gel solutions; and schematic diagram of the coating process of the PAE–AAP hydrogel on the surface of silicone tubing [138]. (**b**) The PPTR hydrogel is composed of an aqueous solution of PBDT and [P4444]Br during the solgel transition [139]. (**c**) Synthesis mechanism and structure of PAM/SA/LiCl antifreeze hydrogel [121]. (**d**) Preparation of the PCM hydrogel [130].

Polyelectrolyte hydrogels have a wide range of applications in flexible electronics, attributed to their distinct conductivity and responsiveness. Unlike polymers, Polyelectrolytes are typically formed by copolymerizing charged monomers with neutral monomers [128]. This copolymerization strategy primarily aims to enhance hydrogel responsiveness to environmental changes by incorporating charged groups and to improve their mechanical properties and sensitivity by forming physical or chemical cross-linking networks [129,130]. Neutral monomers primarily include acrylamide (AAm), N-isopropyl acrylamide (NIPAM), and 2-hydroxyethyl methacrylate (HEMA), which provide the foundational physical structure for the hydrogel [131,132,133]. In contrast, charged monomers, including 2-acrylamide-2-methylpropane sulfonic acid (AMPS), acrylic acid (AAc), 2-(methylacyloxy)ethyl dimethyl-(3-sulfopropyl)ammonium hydroxide (SBMA), and 3-dimethyl(methacryloyloxyethyl)ammonium propane sulfonic acid (DMAPS), induce specific charge characteristics to the hydrogel, enabling the hydrogel to respond to environmental factors such as pH and ionic strength [134,135,136,137]. These strategies not only expand the functional diversity of hydrogels but also significantly enhance their application potential, particularly in sensor technology. For example, Li et al. [138] developed a lubricating antibacterial hydrogel by combining a polyvinylpyrrolidone (PVP) chain with polyacrylamide (P(AAc-co-AAm)). The lactam group in the PVP molecular structure and the amide group in the P(AAc-co-AAm) molecular structure endow the hydrogel coating with good hydrophilicity (WCA ≈ 26.7°) and excellent lubrication performance (COF ≈ 0.129), as shown in Figure 9a. In addition, Zheng et al. [139] developed the first reversible fiber network hydrogel by synthesizing polyelectrolytes, as shown in Figure 9b. In their research, the double helix poly(2,2′-disulfonyl-4,4′-benzidine terephthalamide) (PBDT) and tetrabutyl phosphine bromide ([P4444]Br) exhibited a unique sol–gel transition. [P4444]Br had a lower critical solution temperature (LCST) higher than room temperature, and the water gel was successfully applied as a spray coating on the superhydrophobic vertical Teflon surface.

### 4.3. Inorganic Salt Doping

Compared to polyelectrolyte hydrogel strain sensors, those utilizing inorganic salt gels demonstrate faster response times and higher sensitivity [140]. This modification strategy typically employs physical or chemical methods to directly incorporate specific salts, such as sodium chloride (NaCl), lithium chloride (LiCl), and copper ions (Cu^2+^), into the hydrogel matrix, resulting in ion-doped electrolyte hydrogel sensors with excellent performance [141,142]. Physical doping involves immersing the hydrogel in a solution containing inorganic salts, ensuring uniform dispersion of the salts within the hydrogel matrix to produce ion-doped hydrogels. For example, Zhang et al. [143] developed a polyacrylamide/sodium acrylate/lithium chloride (PAM/SA/LiCl) hydrogel with high frost resistance and conductivity by combining PAM and SA with LiCl (Figure 9c). This hydrogel forms a semi-interpenetrating polymer network (semi-IPN) through hydrogen bonding, enhancing its mechanical properties to achieve a fracture strain of 2100% and a fracture stress of 110 kPa. The inclusion of LiCl significantly boosted the ionic conductivity to 21.7 S/m and improved its strain sensitivity, with a strain coefficient of 17.45. However, this method is highly dependent on salt concentration, which significantly impacts the key performance of the sensor in practical applications. To address this challenge, Huang et al. [144] developed a thixotropic hydrogel with high thixotropy by introducing cellulose nanocrystals (CNC). In their study, it was demonstrated that introducing CNC is beneficial for reducing the effects of NaCl and CaCl_2_ inorganic salt concentrations. Wang et al. [145] developed an inorganic salt ion-enhanced conductive hydrogel using hydroxyethyl cellulose, hydroxyethyl acrylate, lithium chloride, and a glycol/water binary solvent, and further verified the excellent properties imparted by inorganic salt ions. These include self-adhesiveness, high tensile strength, fatigue resistance, frost resistance (−40 °C), and durable moisture retention (80 °C).

### 4.4. Integration of Nanomaterials

The integration of nanomaterials offers an innovative approach to developing CH-FWSs with enhanced mechanical properties and sensing responses. Materials such as gold nanoparticles (AuNPs), gold nanorods (AuNRs), silver nanoparticles (AgNPs), carbon nanotubes (CNTs), GO, and MXenes are incorporated into various polymer networks, through physical doping or chemical cross-linking. This integration not only enhances the sensors’ responsiveness to diverse stimuli but also significantly improves their potential for practical applications by forming dynamic covalent bonds, opening up new opportunities for wearable electronic devices and human health monitoring systems [146,147,148,149]. For example, researchers developed a novel fluorescent nanoprobe structure hydrogel strain sensor that achieves high sensitivity in detecting morphine molecules. This sensor was generated through in situ synthesis, embedding AuNPs and UiO-66 within a PVA hydrogel [150]. The sensor exhibits excellent biocompatibility and safety, along with a wide linear detection range from 0.02 to 2.0 μg/mL and a low detection limit of 0.016 μg/mL, providing an effective tool for biomedical detection.

However, the inherent strong hydrophobicity and limited solubility of hydrogels pose a significant challenge for achieving compatibility with highly hydrophilic hydrogels [151]. To overcome these limitations, Lin et al. [152] developed a non-covalently modified CNT hydrogen-bond cross-linking network of carboxylated styrene butadiene rubber (XSBR) and hydrophilic sericin. This network served as the basis for a multifunctional hydrogel sensor, as shown in Figure 9d. This modification not only significantly improved the dispersion of CNTs within the hydrogel matrix but also enhanced the interfacial interaction between CNTs and the polymer network. Additionally, MXene (Ti3C2Tx) nanosheets have garnered considerable attention in the field of hydrogel strain sensors due to their excellent conductivity and hydrophilicity. For example, Wu et al. [153] developed an innovative dual-network hydrogel flexible strain sensor by combining MXene with PAM and carboxymethyl cellulose. This design leverages MXene’s unique structure and surface functional groups to significantly enhance the conductivity and strain sensing performance of the hydrogel.

Table 2 provides a comprehensive overview of the modified materials, conductivity, hydrogel network, and other key parameters. Although progress has been made in the design and application of modified materials based on CH FWSs, challenges still exist, including the need to improve sensitivity, enhance stability, and increase costs. Future research should focus on exploring emerging materials and combining them with artificial intelligence to optimize sensor performance and promote their widespread adoption in practical applications.

Moreover, in the modification strategies for conductive hydrogel-based flexible wearable sensors, achieving high scalability and cost control in nano-material integration represents a viable approach to enhancing industrialization. For instance, utilizing the self-polymerization properties of polydopamine (PDA), Xu et al. [154] first coated silica particles with PDA, followed by chelating Fe^3+^ on their surfaces to synthesize SiO_2_@PDA-Fe nanoparticles. Subsequently, polyacrylamide (PAM), SiO_2_@PDA-Fe, MXene, and xanthan gum (XG) were blended and cross-linked to produce a multifunctional conductive hydrogel (SiO_2_@PDA-Fe/MXene/XG/PAM). The resulting hydrogel-based strain sensor exhibits high sensitivity (GF = 3.14), a cycle life exceeding 2500 cycles, and stable, reliable long-term operation. This device can capture complex movements of various human joints in real time while outputting stable and clear physiological signals. Additionally, it can function as a wearable medical patch for continuous monitoring of vital signs, including electrocardiograms (ECG) and electromyograms (EMG), thereby providing reliable data support for health monitoring. Most significantly, this low-cost one-pot hybrid process holds immense potential for industrialization. As liquid-phase exfoliation technology matures, MXene costs are projected to drop below $100/kg by 2026.

Liang’s team [155] developed a hard, self-healing nanoconfined hydrogel via polymer entanglement within coplanar nanoconfined regions. This hydrogel exhibits a modulus of 50 MPa and a tensile strength of 4.2 MPa. These hydrogels are prepared by polymerizing high-concentration monomer solutions within fully exfoliated synthetic lithium montmorillonite nanosheet scaffolds and forming macroscopic monodomain structures through shear orientation. Although the resulting physical gel exhibits high modulus, it achieves 100% self-repair efficiency and demonstrates high adhesive shear strength on various substrates. Furthermore, nanoconfined hydrogels enable the incorporation of other nanomaterials, such as MXene nanosheets, between lithium montmorillonite nanosheets to achieve synergistic effects. For example, mixing 1.5 wt% MXene (≈1 μm diameter) with 1.5 wt% lithium montmorillonite forms a nanoconfined Hec-MX-PAAm hydrogel. TEM and EDX analyses revealed that MXene nanosheets were homogeneously distributed within the nanoconfined hydrogel. Consequently, the MXene-doped hydrogel maintains its high strength and stiffness, with an E value of 16 MPa and a UTS of 4.3 MPa. Notably, reduced MXene costs will facilitate large-scale production of hydrogels via the MXene polymer blend method.

Gao et al. [156] prepared a novel multifunctional conductive hydrogel based on the natural polymer methyl cellulose and cellulose nanocrystals via a simple, low-cost strategy. The resulting hydrogel exhibits high transparency, superior tensile properties (663.1%), low-temperature resistance (−23.9 °C), excellent conductivity (2.89 S/m), and outstanding UV shielding performance. The hydrogel-based flexible strain sensor can detect human movements, ranging from subtle to large motions, and demonstrates excellent sensitivity and stability across a wide temperature range. Additionally, multiple flexible hydrogels can be assembled into a 3D sensor array to detect spatial pressure distribution and magnitude. Overall, MXene polymer blends, one-pot hybrid systems, and ion-crosslinked natural polymers currently represent the most industrially promising integrated modification strategies for nanomaterials. These approaches offer advantages such as low cost, high consistency, and easy processing, making them suitable for large-scale deployment of flexible wearable sensors.

**Table 2:** CH-FWSs based on different modification strategies.

Modification Strategy	Conductive Material	Hydrogel Network	Sensitivity (GF)	Stretchability	Conductivity (S/m)	Ref.
Conductive polymer	PPy	Silk/PPy	0.54	-	26	[157]
	PPy	RCF-PPy	2	13.9%	0.8766	[158]
	PANi/Fe^3+^	P(AAm-co-AA)	0.48	145–880%	3.67	[159]
	PANi/CNC	PAAm/HA	3.7	2400%	21.7	[160]
	PPy	AG/SBMA	4.58	510%	0.514	[161]
	PEDOT: PSS/MBA	PAA	3.2	894%	8.06	[162]
Polyelectrolyte	P(AM-AMPS)	PVA/DN	1.57	1282%	3.85	[163]
	AMPS	AAm/VIPS	4.2	604%	0.51	[164]
	Alginate/AAc/Al^2+^	AAV/APS	1.2	1198%	12.5	[165]
	AAc/Fe	P(AAc-co-AAm)	1.3	820%	3.24 ± 0.12	[166]
	DMAPS/MAA	SA/PMAA	-	1353%	-	[167]
	SA/SBMA	AAm/MBAA	4.56	1800%	0.15	[168]
Inorganic salt doping	LiCl	PAM/SA	-	-	21.7	[169]
	LiCl	PVA	-	43–120%	24.29	[170]
	ZnCl_2_/CaCl_2_	Cotton cellulose	0.42	500%	7.49	[171]
	NaCl	PVA	5.98	400%	7.14	[172]
	NaCl	PVA/Pullulan	2.69	800%	10.44	[173]
	LiCl	SBMA/AA/HEMA	2.08	505.54%	2.25	[174]
	Ag/Cu	Gel/Na_2_SO_4_	6.8	732.9%	1.35	[175]
Nanomaterial	CNT/CaCl_2_	AAm/SA	2.83	1821%	0.48	[176]
	AP/AgNPs	PVA/Borax	-	480%	0.39	[177]
	rGO	NIPAM	1.12	400%	4.3	[178]
	AgNWs	PVA	-	-	17.39	[179]
	MXene/Na/Li	PVA/TA	5.42	900%	8.1	[180]
	CNT	PNIPAM	2.15	1800%	0.071	[181]
	AgNW/AgF	PVP/PU	4.8	2700%	83.836	[182]
	MXene	PVA/Aam	1.2	1400%	0.07	[183]

### 4.5. Material Advancement and Detection Limit Evaluation

In recent years, the rapid advancement of conductive hydrogel materials in flexible wearable sensors has significantly enhanced the accuracy and scientific rigor of detection limit assessments. Currently, research primarily evaluates the progress in detection limits systematically from three perspectives: material systems, structural designs, and performance metrics. For instance, Zhao et al. [184], inspired by human skin’s self-healing ability, proposed an innovative gel design strategy integrating interpenetrating polymer networks, multi-level hydrogen bonds, and borate ester bonds. This approach effectively resolved the mutual restriction between the mechanical properties and self-healing properties of hydrogels regarding cross-linking strength and polymer chain mobility, achieving synergistic enhancements in the self-healing, tensile strength, mechanical robustness, and toughness of methacryloyl gelatin gels. Based on this, they developed a self-repairing, highly sensitive methacryloyl gelatin hydrogel strain sensor. It exhibited a gauge factor exceeding 3.28, a minimum detection limit below 0.1%, and nearly complete self-repair of sensitivity post-damage (~100%). This flexible strain sensor has successfully enabled high-sensitivity wearable medical monitoring of various human movements and critical physiological signals (e.g., heart rate and pulse waves). The self-healing flexible sensor, even after damage, demonstrates monitoring capabilities comparable to the original device, underscoring its durability and longevity. This study addresses the key challenges of flexible sensors regarding durability and mechanical robustness. In another study, Song’s team [185] developed a simple construction strategy for modulus gradient ionic conductive hydrogels based on phytic acid-polymer interaction mechanisms, using them to build high-performance pressure sensors. Research demonstrated that the prepared pressure sensor achieved both high sensitivity and a wide pressure detection range, exhibiting excellent low-pressure detection capabilities and high-pressure responsiveness. For acoustic waves of varying frequencies and amplitudes, the hydrogel sensor enables sensitive non-contact responses. For sound, it achieves specific and repeatable recognition. Additionally, the sensor can sensitively detect weak airflow with flow rates as low as 0.2 m/s. Furthermore, the sensor demonstrates sensitive detection and response to physiological signals like pulse and muscle vibrations, as well as finger pressure under high stress. Additionally, Yu et al. [186] developed a novel conductive composite hydrogel and proposed a sensor based on this hydrogel. This sensor exhibits state-independent characteristics, low detection limits (0.5% strain and 25 Pa), high linear dependence, and excellent fatigue resistance (>1000 cycles). Moreover, in practical wearable sensor applications, the sensor can detect various external stimuli and human movements, including speech, writing, joint movements, and even small water droplets, highlighting its potential as a next-generation representative skin sensor.

## 5. Sports Application of CH-FWSs

### 5.1. Human Motion Monitoring

**Figure 10:**
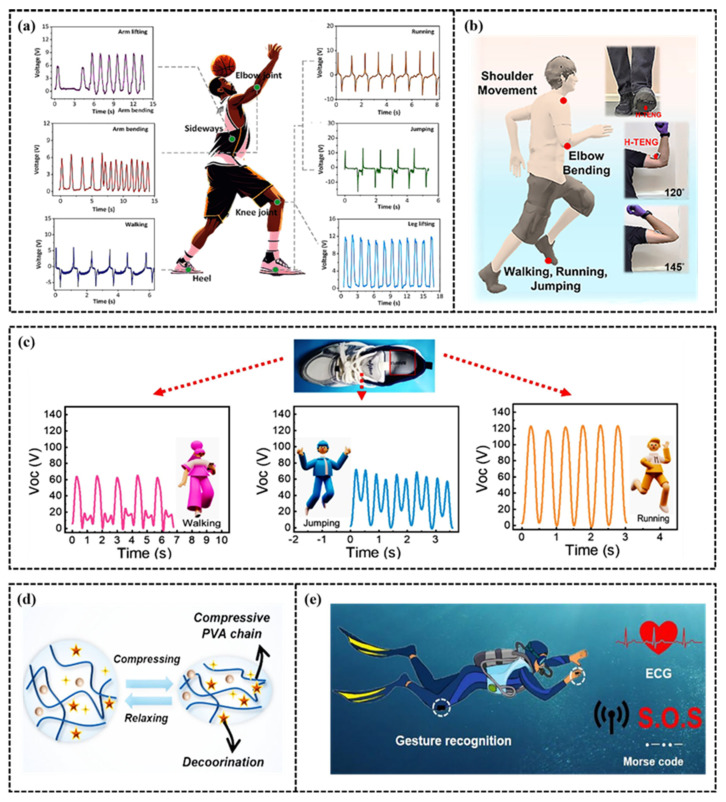
Human motion monitoring based on CH FWSs. (**a**) The output voltage signal of PMN-TENG corresponds to human body parts [188]. (**b**) shows a comprehensive stability assessment of H–TENG open circuit voltage for sensor applications, including detecting body movements such as elbow joint movement, walking, running, and jumping [189]. (**c**) Anti fatigue, self-recovery and anti-fatigue mechanism of LS–LM/PVA hydrogel [190]. (**d**) SC–TENG has potential applications in distinguishing different sports states (such as walking, jumping and running) [191]. (**e**) describes the synthesis process of CMMT using polyethylene glycol (PEG) and polytetramethylene glycol (PTMG) as strain sensors and CMMT based hydrogels, which have excellent sensing ability [192].

CH FWSs exhibit exceptional stretchability and sensitivity, enabling them to adapt to diverse stretching movements during human motion monitoring. Traditional sensors based on organic/inorganic materials, such as carbon nanotubes, graphene, and other conductive materials, typically only achieve approximately 200% stretchability [187]. In contrast, CH FWSs, leveraging their unique structure and performance, can achieve 300% to 10000% stretchability. This capability enables them to accommodate significant stretching during activities such as running, basketball, and swimming, thereby markedly enhancing their ability to monitor human motion. For example, Chen et al. [188] developed a hydrogel-based triboelectric nanogenerator (PMN-TENG) using polydopamine, MXene, and N-isopropylacrylamide for sensor applications. In their study, it was demonstrated that the PMN-TENG not only exhibits exceptional stretchability but also shows excellent conductivity. It was applied to the elbow and knee joints of basketball players for biomechanical energy harvesting and motion monitoring, as shown in Figure 10a. Bablesh et al. [189] developed an innovative stretchable hydrogel by embedding silver nanoparticles (Ag NPs) into a mixture of polyvinyl alcohol (PVA) and sodium nitrate (NaNO_3_). They validated the comprehensive stability of the hybrid-triboelectric nanogenerator (H-TENG) open circuit voltage for sensor applications, including detecting body movements such as elbow joint motion, walking, running, and jumping, as shown in Figure 10b.

However, the tensile properties and sensitivity of conductive hydrogels remain affected after repeated deformation. To address this challenge, for example, Ma et al. [190] enhanced the dispersion and compatibility of lignosulfonate-embedded liquid metal nanoparticles in PVA hydrogels. This improvement bolstered the self-recovery and fatigue resistance of wearable sensors. In their research, they demonstrated that lignin and liquid metal can form a stable shell, ensuring uniform dispersion within the hydrogel. This was evidenced by a compressive strength of 0.53 MPa at 70% strain and a conductivity of 4.87 S m^−1^. These findings further confirm the enormous potential for enhancing human motion tracking and information recognition, as shown in Figure 10c. Presently, it remains a significant challenge to develop hydrogel-based flexible wearable sensors with excellent mechanical properties, adhesion, and conductivity. For this reason, Chen et al. [191] developed a double-network hydrogel using starch/polyacrylamide. They demonstrated that multiple hydrogen bonds impart versatility to the starch/polyacrylamide hydrogel sensor. This versatility enables the sensor to exhibit a wide strain detection range (2580%), high conductivity, and significant adhesion, further verifying its ability to distinguish different human movements, as shown in Figure 10d. Furthermore, the water gel flexible wearable sensor’s extensibility can be affected by swelling during underwater human movement. To meet this challenge, Wang et al. [192] integrated conductive montmorillonite (MMT) into the hydrogel, developing a sensor for underwater monitoring. They demonstrated that the hydrogel maintains excellent extensibility, elasticity, conductivity, and high sensing precision even after prolonged water immersion. This advancement offers a novel approach for promoting underwater intelligent devices, as shown in Figure 10e.

### 5.2. Exercise Electrophysiological Signals

**Figure 11:**
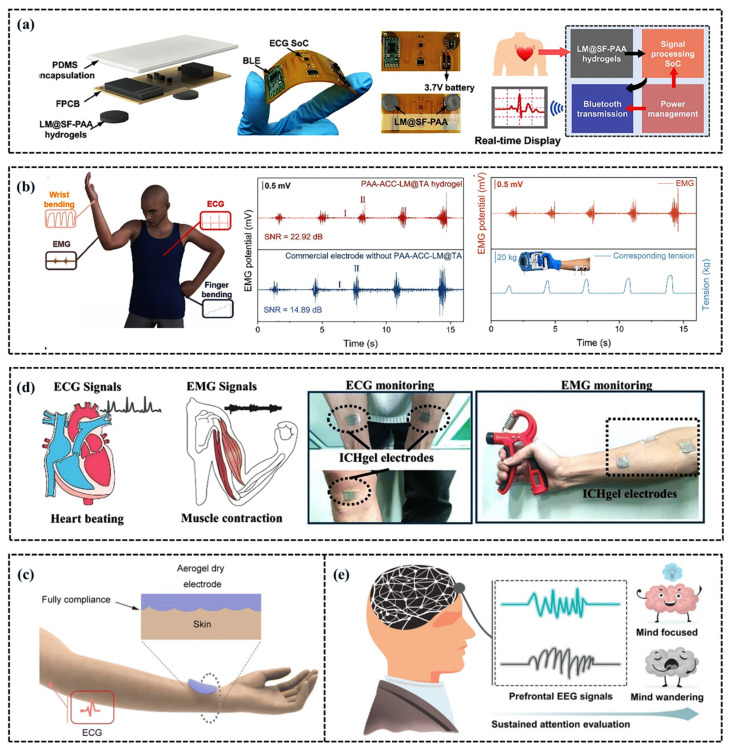
The monitoring of electrophysiological signals of CH FWSs. (**a**) Based on LM@SF-PAA Schematic diagram of portable flexible ECG monitoring patch with hydrogel electrode [197]. (**b**) Multifunctional sensing applications of liquid metal-based hydrogels as electrophysiological electrode substitutes [198]. (**c**) Use the prepared PSG dry electrode to detect epidermal electrocardiogram signals [199]. (**d**) Schematic diagram of EMG and ECG test and photos of ICHgel flexible electrode for EMG and ECG monitoring [200]. (**e**) Application of hydrogel electrode in continuous attention assessment [201].

CH FWSs, as a new generation of monitoring tools, have demonstrated significant potential in tracking physiological changes during exercise. Electrocardiographic signals (ECG), electromyographic signals (EMG), and electroencephalographic signals (EEG), which are monitored during exercise, can assess exercise load, fatigue, and recovery in athletes, while facilitating optimization of training techniques [192,193,194,195]. Real-time monitoring of these physiological signals has established CH FWSs as a powerful tool for exercise physiology monitoring [196]. Their high sensitivity and versatility enable the detection of subtle physiological changes across multiple environments during exercise. To date, CH FWSs have demonstrated multifunctional monitoring applications in sports science. For instance, Yan et al. [197] utilized novel liquid metal@silk protein peptide (LM@SF) core–shell particles to develop a new type of hydrogel flexible sensor. In this sensor, SF not only promotes dispersion of the core LM but also stabilizes free radicals, while enhancing its toughness, adhesion, and conductivity. This demonstrates that the hydrogel can enable continuous wireless monitoring of ECG and EMG during movement and long-term wear, as shown in Figure 11a. Commercial Ag/AgCl gels remain susceptible to interference from the electrode-skin interface during movement. To address this issue, Wei et al. [198] developed a multifunctional hydrogel for electrophysiological sensors using liquid metal TA-coated EGaIn (EGaIn@TA). This hydrogel exhibits unique rheological and adhesive properties that produce a conformal electrode-skin interface, enabling acquisition of stable electrophysiological signals during exercise, including ECG, EMG, as shown in Figure 11b.

However, water gel suffers from sweat-induced limitations during exercise, leading to challenges including poor adhesion and compromised electrical stability. To address these limitations, Gao et al. [199] developed a gel electrode with exceptional skin compliance by combining conductive PEDOT:PSS with surfactants and cross-linking agents, as shown in Figure 11c. They demonstrated that the gel features micron-sized pores and high porosity. Additionally, it exhibits a low compression modulus (<2 kPa) and a low density (10 mg cm^−3^), which collectively enhance its adhesion, permeability, and reduce skin contact impedance, rendering it less susceptible to sweat interference. This enables the acquisition of high-quality ECG signals across diverse scenarios, including sports activities and varying skin conditions (dry/wet). Wu et al. [200] utilized physically interactive ionic conductive hydrogels (ICHgel). Their study demonstrated that the hydrogel’s excellent adhesion and stretchability serve as critical factors between epidermal tissue and flexible devices, enabling stable EMG signal acquisition across different motion amplitudes as shown in Figure 11d. Furthermore, EEG signal acquisition remains susceptible to interference from electrode deformation, sweating, excessive movement, vibration, and environmental factors. To overcome these challenges, Han et al. [201] synthesized a hydrogel network with tissue-mimicking modulus and superior flexibility by integrating polyvinyl alcohol and polyvinyl pyrrolidone. They demonstrated that incorporating polydopamine nanoparticles—generated via polydopamine oxidation—significantly enhances the hydrogel’s transparency, self-adhesiveness, and reduces its impedance, as shown in Figure 11e. This multi-channel, wirelessly operated hydrogel electrode forms a conformal and stable tissue interface, characterized by high channel uniformity, low interfacial contact impedance, minimal power noise, long-term stability, and robust resistance to sweat and movement. With advances in artificial intelligence, these hydrogel-based wearable flexible sensors can wirelessly transmit data to smartphones and other devices, thereby expanding the potential applications of wearable technologies in motion monitoring.

### 5.3. Sports Electrochemical Biological Monitoring

**Figure 12:**
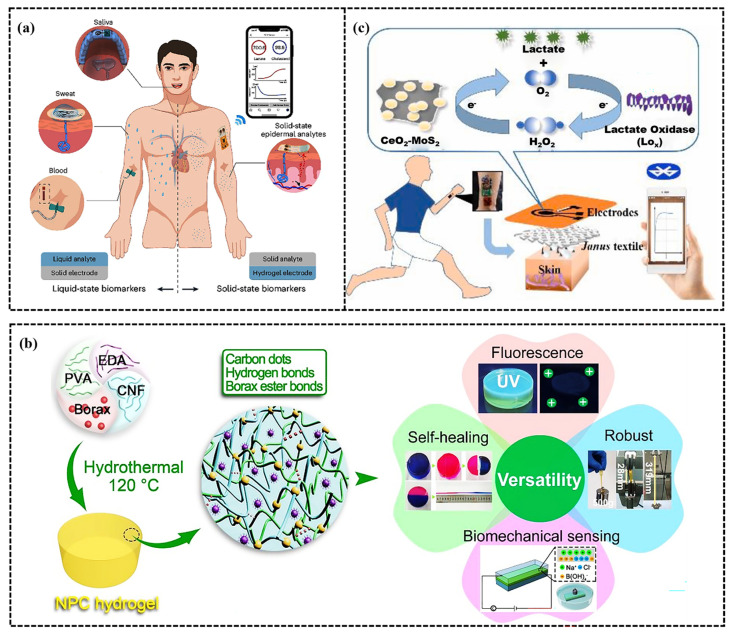
CH FWSs based on electrochemical biomonitoring of movement. (**a**) Various types of biological fluids from different locations (such as saliva, sweat and blood) and SEB [207]. (**b**) shows the preparation materials of NPC hydrogel and the schematic diagram of capacitive sensor for monitoring pressure, strain and glucose concentration [208]. (**c**) Schematic diagram of nano enzyme electrochemical biosensor for monitoring sweat lactic acid [209].

In the realm of sports electrochemical biosensing, CH FWSs offer athletes an innovative alternative to traditional invasive monitoring methods, attributed to their superior biocompatibility, sensitivity, and mechanical properties. Unlike conventional invasive monitoring, these sensors can adhere securely to human skin or biological tissues for non-invasive monitoring, minimizing the risk of injury during the monitoring process [202,203]. The fundamental principle involves integrating electrochemically active materials with biometric components (such as enzymes) to generate quantifiable electrochemical signals, enabling real-time monitoring of exercise-related biochemical indicators, including lactate, glucose, and cholesterol [204,205,206]. For instance, when monitoring glucose during exercise, concentration fluctuations trigger enzymatic reactions within the sensor. These reactions catalyze specific electrochemical processes, producing current changes that reflect variations in glucose levels. Ruth et al. [207] employed an ion-electron double-layer hydrogel sensor to facilitate the continuous dissolution, diffusion, and electrochemical reaction of solid analytes, as shown in Figure 12a. This innovative material design markedly enhances the sensor’s sensitivity, enabling a detection limit of 0.51 nmol mL^−1^ for water-soluble analytes like lactic acid and 0.26 nmol mL^−1^ for water-insoluble analytes such as cholesterol. Compared to traditional hydrogel sensors, this represents a substantial improvement in detection sensitivity, which is vital for real-time monitoring of biochemical indicators during sports activities. The use of advanced materials not only lowers the detection limit but also improves the sensor’s overall performance, enabling non-invasive continuous monitoring in dynamic environments. Wang et al. [208] utilized a one-pot method to synthesize multifunctional hydrogels (NPC) incorporating complex hydrogen bonds, dynamic borate esters, and nitrogen-doped carbon dots. As shown in Figure 12b, they demonstrated that this complex network structure enables the NPC capacitive hydrogel sensor to exhibit excellent linear capacitive responses to pressure, strain, and glucose concentration, establishing multiple linear correlations for real-time quantitative pressure and glucose sensing.

Furthermore, conventional lactate detection methods are invasive. To address this challenge, Weng et al. [209] developed a novel wearable nanoenzyme electrochemical biosensor by integrating molybdenum disulfide (MoS_2_) nanoenzymes into a polymer matrix. This advanced material integration significantly improved the detection range of the sensor, allowing it to detect lactate in artificial sweat within the range of 0.1–50.0 mM. This wide detection range is a direct result of the enhanced material properties of MoS_2_ nanoenzymes, which provide higher sensitivity and specificity compared to conventional materials. This advancement is critical for real-time monitoring of lactate levels during sports activities, as it enables more accurate assessment of athletic performance and recovery. Du et al. [210] developed a flexible hydrogel sensor for monitoring human motion signals and sweat pH/lactic acid using poly(acrylamide-co-acrylic acid)/polyaniline/lithium bromide (PAMAAni/LiBr). Research demonstrates that integrating polyaniline’s conjugated structure with lithium bromide’s ionization principle creates a dual-conductive network. This design confers excellent electrochemical performance on the sensor for pH detection (pH 1–12) and lactate measurement, featuring a broad detection range (0.25–50 mM) and low detection limit (1.98 μM). Currently, most non-invasive cholesterol sensors depend on continuous in situ detection of biomarkers in biological fluids like sweat. However, this approach faces limitations due to restricted access to biological fluids. To address this limitation, Ning et al. [211] prepared an alginate (Alg) hydrogel with luminescent chemiluminescence (CL) for rapid quantitative cholesterol detection. In their research, the luminous CL hydrogel (HRP/COD/Luminol/Alg hydrogel) incorporates Luminol as a chemiluminescent reagent, along with horseradish peroxidase (HRP) and cholesterol oxidase (COD) for enzymatic cascade reactions. The HRP/COD/Luminol/Alg hydrogel exhibits excellent stability, effectively preventing enzyme inactivation during long-term storage.

### 5.4. Commercial Viability

Conductive hydrogels show great application potential in sports wearable sensors, but large-scale commercialization requires further optimization in durability, sweat resistance, and response time. Continuous material modification, structural design, and process innovation are expected to enhance conductive hydrogel performance and meet the high standards of motion monitoring. In practical applications, wearable devices must endure frequent mechanical stresses, including stretching, bending, and compression, while adapting to various environmental conditions. For instance, Zhang et al. [180] developed a double-network hydrogel via physical and chemical crosslinking. It exhibits 900% elongation, excellent fatigue resistance, and can endure 1800 cyclic tensile tests. Leveraging these properties, the hydrogel was integrated into a strain sensor that successfully detects human motion signal changes and recognizes various gestures. This high durability allows the conductive hydrogel sensor to maintain stable long-term performance, satisfying motion monitoring requirements.

Sweating during exercise is common, so sensors require robust sweat resistance. Due to their hydrophilicity, conductive hydrogels readily absorb sweat, affecting performance. However, hydrogel sweat resistance can be enhanced through material modification, such as organic solvent replacement or surface modification. For example, Qin et al. [212] developed an organic hydrogel with self-healing, self-adhesive properties, high sensitivity, and a wide temperature range using bionic component design and organic solvent replacement. The organic hydrogel-based sensor demonstrates excellent reliability and a broad sensing range (5–1500%). It accurately monitors human joint movement, wrist pulses, micro-expressions, and sound signals, aiming to correct athlete posture and enhance training effectiveness. Importantly, it maintains excellent mechanical and electrical properties even under harsh or general environmental conditions (60 days of storage).

In motion monitoring, sensors must respond rapidly to human body movement changes to provide real-time accurate data. For instance, Lu’s research team [213] combined bacterial cellulose (BC) nanofiber networks with polyacrylamide (PAM)/polyvinyl alcohol (PVA) dual networks. By leveraging the NaCl-induced Hofmeister effect to regulate material properties, they developed the PAM/PVA/BC NaCl dual-network hydrogel. The study employed UV-induced in situ polymerization and systematically optimized the BC-to-NaCl ratio, enabling precise control of material properties. The ionic conductive network provides the hydrogel with conductivity up to 1.3 S/m and exceptional strain response. In human joint motion monitoring, PAM/PVA/BC NaCl sensors accurately capture subtle movements like finger bending (0–90°) and wrist rotation. They exhibit a gauge factor of 2.1 and a signal response time of <100 ms. After 8 h of continuous use, resistance fluctuation remains <5%, demonstrating excellent stability and offering more accurate data support for motion analysis.

Key factors driving the commercial viability and market expansion of conductive hydrogel-based wearable sensors include growing demand for personalized and continuous health monitoring, sensor technology iteration, and deep integration with wearable devices [214]. However, conductive hydrogel-based wearable sensors face several commercialization challenges [215]: (1) freezing in low-temperature environments, swelling in wet conditions, and water evaporation in high-temperature/dry settings significantly challenge sensor long-term stability; (2) Most conductive hydrogels lack air permeability, potentially causing skin inflammation during long-term wear; (3) When attached to the body, collisions, scrapes, or wearer movements can generate additional strain signals, severely affecting sensor detection accuracy; (4) The absence of high-precision, standardized manufacturing technologies affects sensor performance consistency and large-scale production feasibility. Thus, developing highly accurate, standardized preparation methods is crucial for the commercialization of multi-functional conductive hydrogel wearable sensors.

## 6. Conclusions and Prospects

As a cornerstone of modern flexible electronic technology, flexible wearable sensors based on conductive hydrogels (CH FWSs) demonstrate distinct potential across multiple domains, including human motion monitoring, health status tracking, and human–computer interaction interfaces. Despite the relative paucity of comprehensive research on CH FWSs in sports monitoring applications over recent years, this article systematically addresses this research gap through an in-depth exploration of their latest scientific advancements and practical application potential.

Current research on conductive hydrogels primarily concentrates on enhancing their performance metrics, including conductivity, self-adhesion, self-healing capabilities, and biocompatibility while actively investigating innovative conductive mechanisms, novel materials, and structural optimization strategies. These investigations strive to optimize pivotal performance parameters, including conductivity, tensile strength, strain operating range, sensitivity, and response time. For instance, while doping conductive materials remarkably enhances hydrogel conductivity, this process may adversely affect their mechanical properties and self-healing efficiency. The self-adhesion and self-repair properties of conductive hydrogels confer substantial application potential in the sports domain. These properties are typically achieved through covalent or non-covalent interactions between material surface characteristics and internal network structures. Nevertheless, preserving material flexibility while enhancing adhesion and self-healing efficiency continues to represent a principal research challenge. Furthermore, the intrinsic biocompatibility of conductive hydrogels has established a foundation for their application in long-term wearable monitoring and implantable medical device development. However, additional research is necessary to ensure these sensors do not elicit rejection reactions in biological tissues during prolonged use and to improve their degradability.

Overall, resistive hydrogel sensors demonstrate significant value for human movement and personal health monitoring in wearable devices attributed to their simple structure, convenient manufacturing, high sensitivity, excellent biocompatibility, flexibility, and robust self-adhesive properties. Capacitive hydrogel sensors exhibit potential applications in flexible touch panels and related fields attributed to their linear response characteristics, rapid response time, and stability under extreme stimuli. Furthermore, piezoelectric-mode hydrogel sensors possess significant advantages in electronic skin and wearable device applications stemming from their simplified design configuration and enhanced functional characteristics. Despite these advancements, conductive hydrogel-based sensors continue to encounter numerous challenges in enhancing linearity, sensitivity, and response time.

Recent research demonstrates that combining conductive polymers, polyelectrolytes, inorganic salt doping, and nanomaterial composite technologies has significantly enhanced the sensitivity, stability, and adaptability of conductive hydrogels. These breakthroughs not only optimize sensor core performance but also create novel opportunities for their multifunctional integration and intelligent applications. However, achieving optimal dispersion of material additives in conductive hydrogels remains a critical challenge in technological development.

Notably, CH FWSs can accurately capture human movements leveraging their exceptional tensile performance, thereby assisting coaches in developing training programs based on scientific data. Furthermore, these sensors can monitor subtle physiological changes, including pulse, blood pressure, electrocardiograms, electromyograms, and electroencephalograms, offering data support for personalized training and rehabilitation treatments. For instance, nanoparticle-enhanced conductive hydrogel sensors can reliably monitor heart rate and pulse under extreme conditions. Additionally, these CH FWSs can real-time monitor sports-related electrochemical biomarkers, including lactate, glucose, and sweat, which are essential for evaluating athletes’ training loads and recovery statuses. Despite their exceptional performance, the stability and accuracy of these sensors require further enhancement to enable dynamic monitoring and applications in specialized environments, including ice, snow, and underwater sports.

Overall, research on CH FWSs is advancing rapidly, primarily emphasizing enhanced practical application efficiency through performance improvements, structural optimizations, and application expansions. Future research should concentrate on material innovation, structural design, and functional integration to thoroughly investigate and address the challenges associated with large-scale production and cost-effectiveness of CH FWSs, thereby facilitating their practical applications in sports. These advancements have established a novel pathway for scientific training and health monitoring applications utilizing CH FWSs. Furthermore, as materials science and intelligent algorithms continue to advance, CH FWSs are anticipated to assume a more pivotal role in sports health applications, offering athletes and the general public more precise, comfortable, and user-friendly monitoring solutions.

## Data Availability

Not applicable.
